# Integrative analysis reveals a clinicogenomic landscape associated with liver metastasis and poor prognosis in hepatoid adenocarcinoma of the stomach

**DOI:** 10.7150/ijbs.71449

**Published:** 2022-08-29

**Authors:** Junjie Jiang, Yongfeng Ding, Jun Lu, Yanyan Chen, Yiran Chen, Wenyi Zhao, Wenfan Chen, Mei Kong, Chengzhi Li, Xiaodong Teng, Quan Zhou, Nong Xu, Donghui Zhou, Zhan Zhou, Haiyong Wang, Lisong Teng

**Affiliations:** 1 Department of Surgical Oncology, The First Affiliated Hospital, Zhejiang University School of Medicine, Hangzhou, China.; 2 Department of Medical Oncology, The First Affiliated Hospital, Zhejiang University School of Medicine, Hangzhou, China.; 3 Institute of Drug Metabolism & Pharmaceutical Analysis & Zhejiang Provincial Key Laboratory of Anti-Cancer Drug Research, College of Pharmaceutical Sciences, Zhejiang University, Hangzhou, China.; 4 Department of Pathology, The First Affiliated Hospital, Zhejiang University School of Medicine, Hangzhou, China.; 5 Institute of Immunology, Zhejiang University School of Medicine, Hangzhou, China.

**Keywords:** hepatoid adenocarcinoma of the stomach, liver metastasis, prognosis, whole-exome sequencing, clinicogenomic landscape

## Abstract

Hepatoid adenocarcinoma of the stomach (HAS) is a rare subtype of gastric cancer (GC) that histologically resembles hepatocellular carcinoma (HCC). Despite its low incidence, HAS had a poor 5-year survival rate. Currently, the linkages between clinicopathological and genomic features of HAS and its therapeutic targets remain largely unknown. Herein, we enrolled 90 HAS patients and 270 stage-matched non-HAS patients from our institution for comparing clinicopathological features. We found that HAS had worse overall survival and were more prone to develop liver metastasis than non-HAS in our cohort, which was validated via meta-analysis. By comparing whole-exome sequencing data of HAS (n=30), non-HAS (n=63), and HCC (n=355, The Cancer Genome Atlas), we identified a genomic landscape associated with unfavorable clinical features in HAS, which contained frequent somatic mutations and widespread copy number variations. Notably, signaling pathways regulating pluripotency of stem cells affected by frequent genomic alterations might contribute to liver metastasis and poor prognosis in HAS patients. Furthermore, HAS developed abundant multiclonal architecture associated with liver metastasis. Encouragingly, target analysis suggested that HAS patients might potentially benefit from anti-ERBB2 or anti-PD-1 therapy. Taken together, this study systematically demonstrated a high risk of liver metastasis and poor prognosis in HAS, provided a clinicogenomic landscape underlying these unfavorable clinical features, and identified potential therapeutic targets, laying the foundations for developing precise diagnosis and therapy in this rare but lethal disease.

## 1. Introduction

Hepatoid adenocarcinoma of the stomach (HAS) is a rare subtype of gastric cancer (GC) that histologically resembles hepatocellular carcinoma (HCC), accounting for 0.38-1.6% of all GC 1. HAS was recognized as a highly malignant carcinoma, featuring rapid progression, high liver metastasis propensity, and poor prognosis 2, 3. Limited by a scarcity of large-scale clinical research and a critical knowledge gap regarding genomic features in HAS, little consensus on its standardized diagnostic and therapeutic strategies has been achieved. Currently, under standard management for conventional gastric cancer (CGC), the 5-year survival rate of advanced-stage HAS patients was only 9% 2.

Since firstly reported in 1985 4, several HAS cases have been reported worldwide, primarily in China 5 and Japan 6. Due to extremely similar clinicopathological features between metastatic HAS and HCC, such as elevated serum alpha-fetoprotein (AFP) level and metastatic liver lesion mimicked HCC-like morphologically, metastatic HAS patients were easily misdiagnosed as HCC in clinical practice 7, 8. Controversially, most case reports revealed that HAS patients had an inferior prognosis than non-HAS patients 5, 9, 10, whereas a few studies did not observe a significant difference of prognosis between HAS and non-HAS patients^11^, ^12^. So far, most clinical studies focusing on HAS were limited to case reports, case series, or small-sample studies. Therefore, a large-scale study with systematic analysis is urgently required to investigate and validate the clinicopathological characteristics and prognosis of HAS.

Characterization of molecular landscape of HAS is a crucial step to deepen the understanding of its clinicopathological features and develop precise therapeutic strategies. Nevertheless, current evidence regarding the molecular characteristics of HAS was mainly established on a limited number of studies. A previous study identified a list of recurrently mutated genes and copy number gains in HAS using targeted sequencing on a panel of 483 cancer-related genes 13. A recent multi-omics study demonstrated the molecular features associated with hepatoid differentiation in a non-metastatic HAS population 14. Despite recent advances in molecular characterization, the molecular mechanism underlying tumor metastasis and unfavorable prognosis in HAS remains unclear.

Herein, we constructed a large clinical HAS cohort and performed comparative analysis and meta-analysis to uncover the distinct clinicopathological characteristics and prognosis of HAS compared with non-HAS. Then, using whole-exome sequencing (WES) analysis, we compared multidimensional genomic features between HAS, non-HAS, and HCC. Importantly, we performed potential targets screening in HAS. These data provide a comprehensive clinicogenomic landscape of HAS, which deepen the understanding of how its unfavorable clinical features are linked to the genomic profiles. In addition, we also identified potential therapeutic targets in this rare but lethal disease.

## 2. Methods

### 2.1 Patient cohort and data collection

This study was approved by the Institutional Review Board of the First Affiliated Hospital of Zhejiang University School of Medicine and conducted in compliance with the guidelines of the Declaration of Helsinki. A total of 12622 GC patients were screened from patients who received biopsies/surgery and underwent standardized pathological diagnoses in the First Affiliated Hospital, Zhejiang University School of Medicine from January 2009 to June 2020. Based on WHO classification of tumors of the digestive system (2019)^15^, HAS was morphologically defined as a tumor composed of large polygonal eosinophilic neoplastic cells (hepatoid differentiation), regardless of the percentage of hepatocyte-like regions or serum AFP level. Two certificated pathologists (M.K. and C.Z.L.) independently confirmed the pathological diagnoses. Any discrepancies were then resolved by consulting another experienced pathologist (X.D.T.). This study enrolled 90 HAS patients and 973 CGC patients with annotated clinicopathological and follow-up data. Subsequently, 270 non-HAS patients were screened from CGC via a manner of 3:1 stage-matched with HAS for comparative analysis. All patients provided written informed consent.

We obtained clinicopathological information from patient medical records in our institutional database. The clinical information included gender, tumor size, tumor location, serum levels of AFP, carcinoembryonic antigen (CEA) and carbohydrate antigen 19-9 (CA199), first-metastasis site, computed tomography (CT) images, and therapy. Pathological features such as TNM stage (American Joint Committee on Cancer (AJCC), 8th edition), vascular invasion, hematoxylin & eosin (H&E)-stained micrographs, and ERBB2 immunohistochemistry (IHC) results were also collected. The baseline clinicopathological characteristics were displayed in Table 1 and Table S1. Patients were followed up via telephone, letters, and medical records. Overall survival (OS) time was defined as the interval from the date of diagnosis to the date of death or the last follow-up point. During follow-up, the site and time of the first metastasis were recorded. Synchronous metastasis was defined as distant metastasis at diagnosis or within six months during follow-up, whereas metachronous metastasis occurred after six months during follow-up 16. We retrospectively collected fresh-frozen tumor tissues with paired tumor adjacent normal tissues from 30 HAS cases and 63 non-HAS cases for WES. In addition, WES data with annotated clinicopathological information of 355 TCGA patients diagnosed as HCC (TCGA-LIHC) were downloaded from the cBioportal database (https://www.cbioportal.org/).

### 2.2 Meta-analysis

We conducted a systematic literature search in PubMed, Web of Science, Embase, Scopus, Cochrane Library, and CNKI database up to January 2021, following the Preferred Reporting Items for Systematic Reviews and Meta-Analyses (PRISMA) guidelines. The search terms were as follows: (“adenocarcinoma” OR “adenocarcinomas” OR (“malignant” AND “adenoma”) OR “malignant adenoma”) AND “hepatoid” AND (“Stomach” OR “Stomachs” OR “Gastric”). We also searched articles in the references if they were potentially relevant to this topic. Inclusion criteria were as follows: (1) pathologically confirmed HAS; (2) studies comparing the difference of survival outcomes between HAS and non-HAS patients; (3) the availability of reported survival data; and (4) full-text articles published in English or Chinese. Exclusion criteria included (1) cases reports, case series, reviews, meta-analyses, letters to the editor, conference abstracts, and comments, and (2) studies without non-HAS/CGC patients as control. Finally, five eligible studies were retrieved from online databases 2, 3, 11, 12, 17. Additionally, the current study containing 90 HAS and 270 stage-matched non-HAS patients (named as ZJU cohort) was also included in the meta-analysis. The extracted information included the following items: (1) the first author's name; (2) publication year; (3) country; (4) number of patients; (5) diagnosis criteria for HAS; (6) clinicopathological features including age, gender, T stage, liver metastasis, vascular invasion, and lymph node metastasis; (7) data extraction; (8) information of stage-matched manner; and (9) survival outcomes.

The meta-analysis was performed using R software (version 3.6.1, Missouri, USA). The outcomes included prognosis, liver metastasis, vascular invasion, and lymph node metastasis. Sensitivity analysis was performed to explore the stability of meta-analysis. Statistical heterogeneity was quantified by I^2^, Tau^2^, and Q-statistic. The value of I^2^ represented the heterogeneity level as follows: low (I^2^ < 25%), moderate (I^2^ = 25-75%), or high (I^2^ > 75%). A random-effects model was adopted for meta-analysis. The publication bias was visualized by funnel plot and estimated using Begg's and Egger's tests.

### 2.3 SEER database analysis

Clinicopathological and follow-up data of patients from the Surveillance, Epidemiology, and End Results (SEER) database were obtained using SEER*Stat 8.3.9 software. By screening the SEER Research Data,18 Registries, Nov 2020 Sub [2000 - 2018] dataset, GC patients were included according to the following criteria: (I) primary site-labeled: C16.0-Cardia, NOS; C16.1-Fundus of stomach; C16.2-Body of stomach; C16.3-Gastric antrum; C16.4-Pylorus; C16.5-Lesser curvature of stomach NOS; C16.6-Greater curvature of stomach NOS; C16.8-Overlapping lesion of stomach; C16.9-Stomach, NOS; (II) known distant metastasis status/site; and (III) available follow-up information. HAS patients were identified according to the item of ICD-O-3 Hist/behav of 8576/3-hepatoid adenocarcinoma. For clinical comparative analysis, 31 HAS and 100208 non-HAS patients were enrolled in the independent validation cohort (SEER cohort).

### 2.4 Development and validation of a Nomogram for predicting liver metastasis in HAS patients

Aiming to establish a Nomogram model for early predicting liver metastasis in HAS patients, we retrospectively collected and analyzed the clinicopathological information of 90 HAS patients who were diagnosed in our hospital from January 2009 to June 2020. The clinicopathological variables included age, gender, tumor size, tumor location, vascular invasion, lymph node metastasis, tumor differentiation, serum level of alpha-fetoprotein (AFP), carcinoembryonic antigen (CEA), carbohydrate antigen 19-9 (CA19-9), carbohydrate antigen 125 (CA125), serum ferritin (SF), white blood cell (WBC), neutrophil (NEUT), lymphocyte (LYM), neutrophil-lymphocyte ratio (NLR), monocyte (MO), albumin (ALB), globulin (GLB), albumin-globulin ratio (AGR), alanine aminotransferase (ALT), aspartate aminotransferase (AST), alkaline phosphatase (ALP), total bilirubin (TBil), and direct bilirubin (DBil). After removing 13 cases with incomplete clinicopathological information, a total of 77 HAS patients were enrolled in the training cohort. Furthermore, 25 patients diagnosed in our hospital from July 2020 to December 2021 were enrolled in the validation cohort. The chi-square test or Fisher's exact test was performed to evaluate the differences in the baseline clinicopathological variables between the training cohort and the validation cohort. In the training cohort, patients were divided into two subgroups according to the status of liver metastasis. Thereafter, univariate analysis was performed to screen the risk factors associated with liver metastasis. A *P* < 0.10 was adopted as the threshold. Based on these candidates, multivariate logistic regression analysis was performed to construct a predictive model for liver metastasis. According to the regression coefficients, a nomogram was generated with the R package “rms”. The performance of the Nomogram was assessed using receiver operating characteristic (ROC) analysis and calibration curve analysis in both the training cohort and the validation cohort. The area under the ROC curve (AUC) was calculated to assess the predictive accuracy for liver metastasis in HAS patients. The calibration curve analysis was performed using the internal validation with 1000 bootstrap resamples.

### 2.5 DNA extraction and whole-exome sequencing

Genomic DNA from GC tissues and matched normal gastric mucosa was isolated using QIAamp DNA Mini Kit (Qiagen) following the manufacturer's protocol. DNA quality control included monitoring degraded/contaminated DNA on 1% agarose gels and quantifying DNA concentration using Qubit® DNA Assay Kit in Qubit® 2.0 Flurometer (Invitrogen, USA). WES library was prepared using Agilent SureSelect Human All Exon V6 Kit (Agilent Technologies, Santa Clara, CA, USA) following the manufacturer's instructions. Libraries were sequenced on Illumina Hiseq platform (Illumina, San Diego, California, USA) and 150 bp paired-end reads were generated. High-quality clean data were obtained by removing low-quality reads, reads containing an adapter or poly-N. The clean reads were aligned to GRCh37 by BWA v.0.7.8 18.

### 2.6 Somatic mutation and mutational signature analysis

Somatic single-nucleotide variants (SNVs) was identified using muTect (v 1.1.4) 19, and the somatic InDels were detected using Strelka (v1.0.13) 20. Following that, variant call format files were annotated by ANNOVAR 21. Somatic mutation types included missense mutation, splice site, nonsense mutation, frameshift del, in frame del, in frame ins, frameshift ins, and multihit. Frequently mutated gene was identified according to gene mutation frequency (> 10%). Significantly mutated genes were identified using MutSigCV (v1.4) algorithm with a q-value < 0.05 as the threshold. Oncoplot displaying mutational landscape was visualized using R package ComplexHeatmap 22. The mutational signature was decomposed using the Bayesian nonnegative matrix fraction (NMF) algorithm 23. Firstly, somatic mutation variants were divided into 96 trinucleotide mutation contexts according to the mutant base substitutions and the adjacent 3' and 5' flanking nucleotides. Then the Bayesian NMF algorithm was applied to deconstruct the mutational signatures. R package DeconstructSigs 24 was used for signature assignment in each sample of HAS, non-HAS and TCGA-LIHC. Cosine similarity was selected as a metric to compare the similarities of estimated mutational signatures to predefined signatures in Catalogue of Somatic Mutations in Cancer (COSMIC). COSMIC mutational signatures (version 2.0) were used as reference signatures. Cosine similarity > 0.80 was set as the threshold.

### 2.7 Copy number analysis

Based on paired tumor-normal WES data, we determined copy number variation (CNV) using CNVkit with default parameters 25. After that, GISTIC2.0 algorithm was applied to identify genome regions with a significant frequency of CNV 26. Both low-level (GISTIC score, +/-1) and high-level (GISTIC score, +/-2) were adopted as the thresholds to define gene-level CNV. High-level (+/-2) CNV was considered as amplification/deletion. Venn analysis was conducted to identify common and specific CNV between HAS, non-HAS, and TCGA-LIHC.

### 2.8 Pathway enrichment analysis

By integrating somatic mutation and CNV data, we performed pathway enrichment analysis to identify frequently altered pathways of HAS compared with non-HAS using the DAVID tool (https://david.ncifcrf.gov/). Kyoto Encyclopedia of Genes and Genomes (KEGG) pathway was adopted as the gene set for enrichment analysis. The significance of pathway enrichment analysis was determined using a hypergeometric test (FDR < 0.05).

### 2.9 Clonal architecture analysis

Variant allele frequencies (VAFs) and CNV data were processed using multiple bioinformatic pipelines including SciClone 27, PyClone 28, and MOBSTER^29^ to estimate the number of clones in HAS and non-HAS samples. Mutations with VAF greater than 0.8 were excluded to filter out germline mutations. To ensure statistical robustness, only those variants supported by ≥ 100 reads were considered for clonal analysis. Finally, the predicted number of clones detected in the given sample was determined. After repeating the above procedures for all samples, the predicted numbers of clones were compared between HAS and non-HAS.

### 2.10 Evaluation of potential targets and therapeutic efficacy

The gene list of potential targets was downloaded from OncoKB database (https://www.oncokb.org/). Frequently altered targets (≥ 10%) in HAS were screened out and matched with non-HAS for further comparison, and then visualized using R package ComplexHeatmap 22. In this study, three HAS patients received anti-ERBB2 therapy and five HAS patients received anti-PD-1 therapy. Serum AFP and CEA levels of each patient were recorded before and after treatment, and the change percent (CP) was determined as follow: CP (%) = (serum AFP or CEA levels before treatment - serum AFP or CEA levels after treatment) / serum AFP or CEA levels before treatment *100%. The values of 20 ng/mL and 5 ng/mL are defined as the serum AFP and CEA levels threshold, respectively. For each patient with elevated AFP/CEA level before treatment, 65% was defined as the cutoff for response to anti-ERBB2/anti-PD-1 therapy, which is inapplicable for a patient with a normal AFP/CEA level before treatment in this study.

### 2.11 H&E and IHC staining

H&E and IHC staining were performed on 4 μm-thick FFPE tumor tissue sections. For IHC staining, sections were deparaffinized, rehydrated, subjected to antigen retrieval, and blocked by endogenous peroxidase. Following that, the sections were incubated with anti-ERBB2 monoclonal primary antibody (Cell Signaling Technology, #2165), followed by a 30-min incubation with secondary antibody. Staining was visualized using streptavidin-biotin peroxidase complex method (Lab Vision, Fremont, CA, USA). ERBB2 expression in IHC was based on staining intensity in GC cells, and scored using Hofmann's criteria as follows: - (negative), + (negative), ++ (equivocal), or +++ (positive) 30. IHC scoring process was independently performed by two certificated pathologists (M.K. and C.Z.L.). Another experienced pathologist (X.D.T.) was consulted to resolve discrepancies.

### 2.12 Statistical analysis

Statistical analyses were performed using SPSS software (version 21.0) and R software (version 3.6.1). The differences between two categorical variables were examined by chi-squared (χ^2^) test or Fisher's exact test where appropriate. The Mann-Whitney test was used to analyze ranked variables. Kaplan-Meier curves were examined using a log-rank test. The hazard ratio (HR) with corresponding 95% confidence interval (CI) was determined using Cox regression analysis. The odds ratio (OR) with corresponding 95% CI were determined using univariate logistic regression analysis. Two-sided *P*-values were used throughout the analyses. A *P* < 0.05 was considered statistically significant.

## 3. Results

### 3.1 HAS had highly malignant clinicopathological characteristics and a poor prognosis

**Figure 1:**
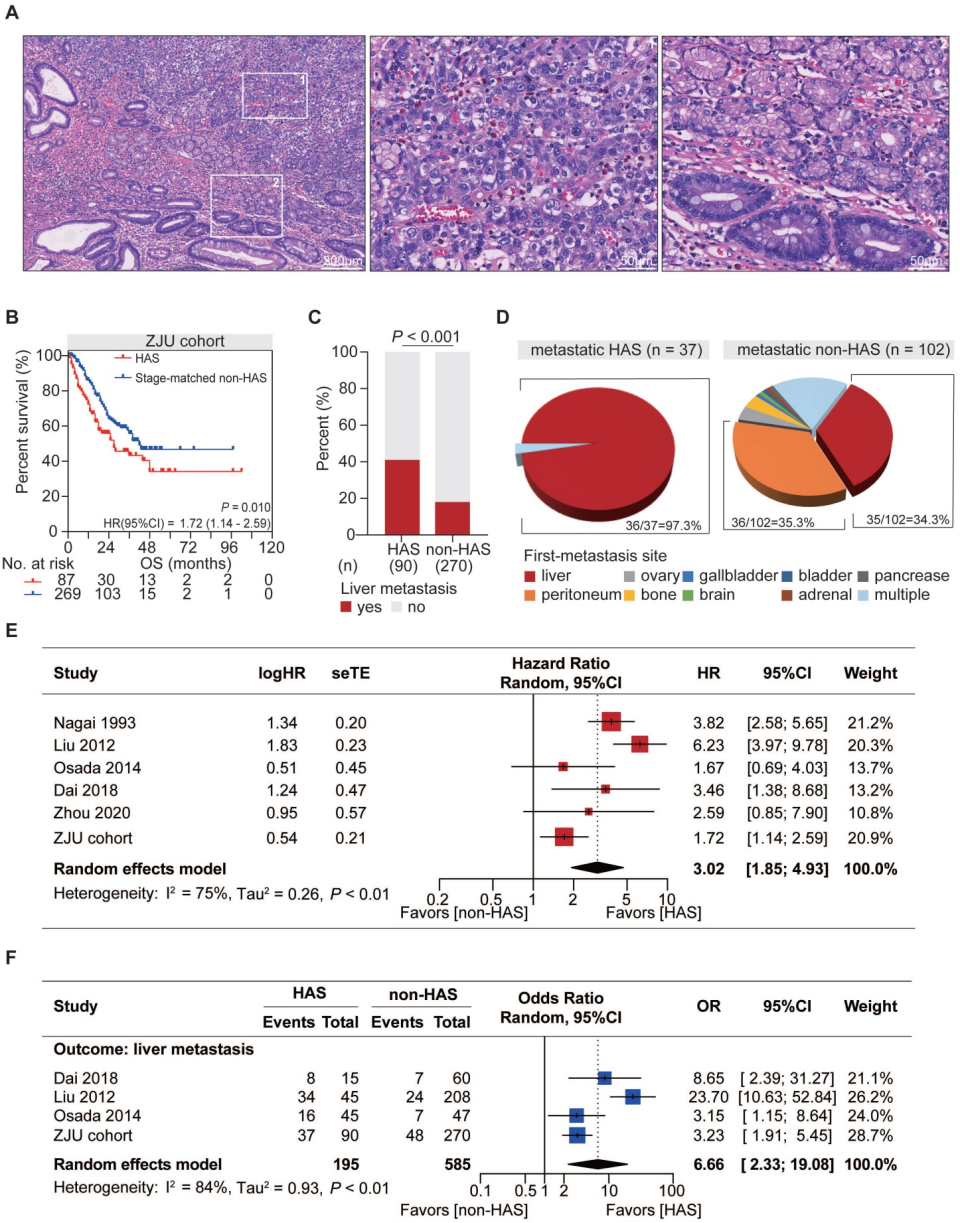
** Distinctive clinicopathological characteristics of HAS in comparison with non-HAS. (A)** Representative H&E-stained micrographs of HAS. The left panel presents the overall histologic features (100X, scale bar 200 μm). The zone “1” and “2” represent the area of hepatoid differentiation (400X, scale bar 50 μm) and tubular adenocarcinoma differentiation (400X, scale bar 50 μm), respectively. **(B)** Comparison of overall survival between HAS and stage-matched non-HAS patients in ZJU cohort. **(C)** Comparison of liver metastasis rate between HAS and non-HAS patients in ZJU cohort.** (D)** Distribution of first-metastasis site in metastatic HAS and metastatic non-HAS patients. **(E)** Forest plot showing the meta-analysis of the hazard ratio for overall survival in HAS versus non-HAS patients. The hazard ratios in each study are presented and the horizontal lines indicate the 95% confidence intervals. (**F**) Forest plot showing the meta-analysis of the odds ratio for liver metastasis in HAS versus non-HAS patients. The odds ratios in each study are presented and the horizontal lines indicate the 95% confidence intervals. HAS, hepatoid adenocarcinoma of the stomach; non-HAS, non-hepatoid adenocarcinoma of the stomach (conventional gastric adenocarcinoma without hepatoid differentiation); OS, overall survival; HR, hazard ratio; OR, odds ratio; CI, confidence interval.

As a unique subtype of GC, HAS exhibited typical pathological features distinct from those of CGC but similar to those of primary HCC. Tumor cells in hepatoid differentiation area harbored classic hepatocyte-like features, such as abundant eosinophilic cytoplasm, large and ovoid nucleus, prominent nucleoli, and sinusoidal vascular channels, all of which were absent in tubular adenocarcinoma component (Figure 1A).

From a clinical point of view, compared with CGC patients, HAS patients showed higher rate of distant metastasis (*P* < 0.001; Table S1), more advanced TNM stage at initial diagnosis (*P* < 0.001, Table S1), and significantly worse OS (HR = 3.13, 95%CI = [2.00, 4.89, *P* < 0.001; Figure S1). To reduce bias from TNM stage for further comparison between HAS and non-HAS, 270 cases of non-HAS were screened from CGC via a manner of 3:1 stage-matched with HAS for comparative analysis. Compared with non-HAS, HAS revealed a higher rate of vascular invasion, higher serum AFP and CEA levels, and more abundant ERBB2 expression in IHC (Table 1). Notably, survival analysis revealed that HAS patients had more unfavorable OS than stage-matched non-HAS patients (HR = 1.72, 95%CI = [1.14, 2.59], *P* = 0.010; Figure 1B). Multivariate Cox analysis further identified hepatoid differentiation as an independent risk factor (HR = 1.54, 95%CI = [1.06, 2.24], *P* = 0.023; Figure S2) when adjusted for age, gender, and TNM stage in ZJU cohort. Remarkably, liver metastasis occurred more frequently in HAS than non-HAS patients (41.1% vs 17.8%, *P* < 0.001; Figure 1C). More importantly, comparative analysis for the first-metastasis site demonstrated a dominantly different metastasis pattern between metastatic HAS and non-HAS (Figure 1D). To be specific, most metastatic HAS only developed liver metastasis (97.3%), whereas metastatic non-HAS developed multi-organ metastases, including peritoneum (35.3%), liver (34.3%), ovary (3.9%), bone (3.9%), gallbladder (1.0%), brain (1.0%), bladder (1.0%), adrenal (1.0%), pancreas (1.0%), and multiple sites (17.6%). In addition, given the information of American population from SEER database, it was validated that HAS patients had worse OS and developed a higher tendency of liver metastasis (Figure S3), consistent with those findings in ZJU cohort (Figure 1B-D).

Meta-analysis was conducted to validate the distinctive clinical characteristics of HAS compared with non-HAS patients. A systematic search in online databases was conducted to identify eligible studies (Figure S4). Finally, the meta-analysis included five published studies 2, 3, 11, 12, 17 and our cohort with 1812 patients, including 278 HAS and 1534 non-HAS patients (Table S2-3). Meta-analysis revealed that HAS patients harbored significantly worse OS (pooled HR = 3.02, 95%CI = [1.85, 4.93], *P* < 0.001; I^2^ = 75%, random effect; Figure 1E) and developed more recurrent liver metastasis (pooled OR = 6.66, 95%CI = [2.33, 19.08], *P* < 0.001; I^2^ = 84%, random effect; Figure 1F) comparing to non-HAS, consistent with those findings in ZJU cohort (Figure 1B-C) and SEER cohort (Figure S3). Sensitivity analyses and publication bias tests indicated that pooled results were robust without significant publication bias (Figure S5). The heterogeneity might be partially explained by different diagnosis criteria between studies. In detail, in the subgroup using morphology with or without AFP production as diagnosis criteria for HAS, HAS patients both harbored worse OS than non-HAS patients, where the heterogeneity in the subgroups was not significant (morphology: HR =1.89, 95%CI = [1.34, 2.67], *P* < 0.001; I^2^ = 0%, *P* = 0.38; morphology and AFP production: HR = 4.46, 95%CI = [2.91, 6.83], *P* < 0.001; I^2^ = 45%, *P* = 0.16; Table S4).

### 3.2 Identification of a Nomogram model for predicting liver metastasis in HAS patients

HAS harbored a high tendency of liver metastasis, leading to a poor prognosis of patients in clinical practice. Aiming to early predict liver metastasis in HAS patients, we further establish a Nomogram model based on clinically actionable indexes. The training cohort and validation cohort contained 77 and 25 HAS patients with annotated clinicopathological information, respectively. The difference in the baseline characteristics of HAS patients was not significant between the training cohort and validation cohort (Table S5). Then we compared the clinicopathological characteristics between patients with or without liver metastasis in the training cohort.

As a result, six variables were identified as the risk factors associated with liver metastasis in HAS patients (Table S6) when a *P* < 0.10 was adopted as the threshold, including age (*P* = 0.056), serum level of AFP (*P* = 0.075), CA19-9 *(P* = 0.007), CA125 (*P* = 0.025), GLB (*P* = 0.025), and ALP (*P* = 0.001). A Nomogram model based on these variables was established using multivariate Logistic regression analysis (Table S7, Figure S6A). ROC analysis showed that the predictive efficacy of the Nomogram model was powerful and robust in the training cohort (AUC = 0.821, Figure S6B) and validation cohort (AUC = 0.728, Figure S6C). Calibration analysis showed a good coincidence between the predicted value and the actual value of liver metastasis rate for the Nomogram model (Figure S6D-E). Taken together, this study provided an effective tool for clinicians to stratify the risk of liver metastasis in HAS patients, laying a theoretical foundation for early clinical intervention and prognosis guidance.

### 3.3 HAS presented a genomic landscape featured by frequent somatic mutations

**Figure 2:**
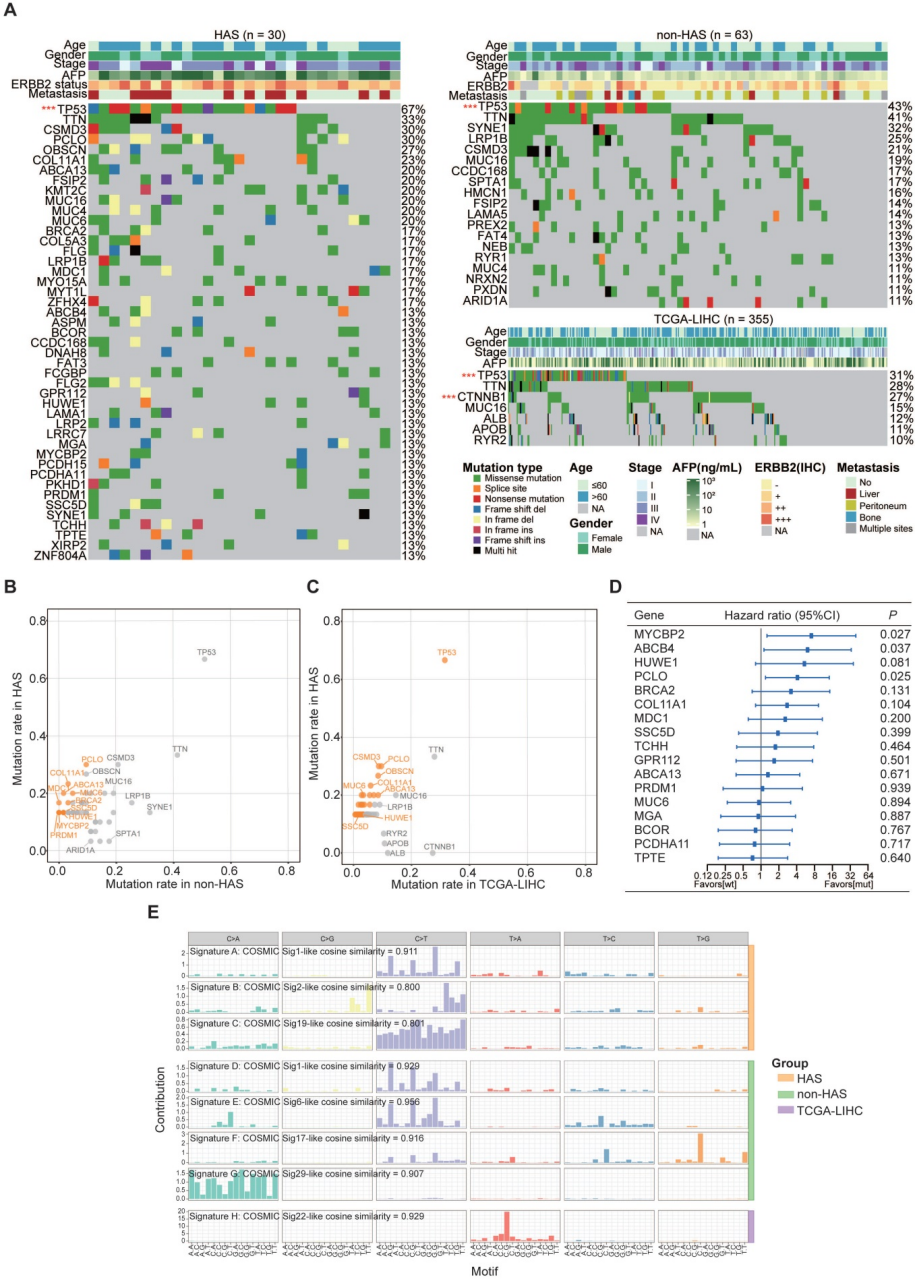
** The landscape of somatic mutations and mutational signatures in HAS, non-HAS, and TCGA-LIHC. (A)** Mutational spectrum of frequently mutated genes identified in HAS, non-HAS, and TCGA-LIHC. Significant mutated genes were identified using MutSigCV and labeled with red star. ^***^P < 0.001. **(B-C)** Gene mutation rates of HAS in comparison with non-HAS **(B)** or TCGA-LIHC **(C)**. Orange dots, genes with significantly higher mutation rate in HAS. **(D)** Association of frequently mutated genes with overall survival in HAS. The hazard ratios of genes are presented and the horizontal lines indicate the 95% confidence intervals. **(E)** Mutational signatures in HAS, non-HAS, and TCGA-LIHC were identified using the Bayesian NMF algorithm. The vertical axis represents the estimated mutation contribution to a specific mutation type based on COSMIC signatures. The horizontal axis represents mutation patterns of 96 substitutions plotted in different colors with denoted order. TCGA-LIHC, hepatocellular carcinoma from TCGA database; wt, wild type; mut, mutation; COSMIC, Catalogue of Somatic Mutations in Cancer database; sig, mutational signature.

Somatic mutations called from HAS and non-HAS were identified based on the raw WES data. WES data of TCGA-LIHC were available from the cBioportal database. The average sequencing depth on target for tumors and normal samples are listed in Table S8 (HAS: 172X and 120X, respectively; non-HAS: 321X and 175X, respectively). All HAS samples contained 38146 somatic mutations with a median of 629 nonsynonymous mutations per tumor, whereas non-HAS contained 49564 somatic mutations with a median of 417 nonsynonymous mutations per tumor. Subsequently, frequently mutated genes (> 10%) identified in HAS, non-HAS, and TCGA-LIHC were depicted in the oncoplot as presented in Figure 2A. A number of frequently mutated genes was screened in HAS comparing to non-HAS and TCGA-LIHC, such as mutation in *PCLO* (30% vs 9.5%, *P* = 0.017, Figure 2B and Table S9; 30% vs 9.6%, *P* = 0.003, Figure 2C and Table S10), *MUC6* (20% vs 4.8%, *P* = 0.029, Figure 2B and Table S9; 30% vs 3.4%, *P* = 0.001, Figure 2C and Table S10), and *BRCA2* (16.7% vs 3.2%, *P* = 0.034, Figure 2B and Table S9; 16.7% vs 3.1%, *P* = 0.005, Figure 2C and Table S10). Besides, although *TP53* mutations were common, the highest frequency was observed in HAS comparing to non-HAS and TCGA-LIHC (66.7% vs 50.8% vs 31.5%; Figure 2B-C and Table S9-10). Furthermore, survival analysis identified several mutated genes associated with poor prognosis in HAS (Figure 2D), including *MYCBP2* (HR = 7.26, 95%CI = [1.26, 39.71], *P* = 0.027), *ABCB4* (HR = 6.09, 95%CI = [1.12, 33.18], *P* = 0.037), and *PCLO* (HR = 4.11, 95%CI = [1.19, 14.16], *P* = 0.025). Patients with *HUWE1* mutation also tended to show worse OS than those with wild type (HR = 5.39, 95%CI = [0.81, 35.98], *P* = 0.081). Interestingly, four HAS patients with *MYCBP2* or *HUWE1* mutation all developed liver metastasis (Figure S7). In addition, we applied Bayesian NMF algorithm to identify the mutational signatures in HAS, non-HAS and TCGA-LIHC and compared the similarity between these signatures and predefined COSMIC signatures. Using a cosine similarity > 0.80 as the threshold, we identified three principal mutational signatures (Signature A-C) in HAS, four (Signature D-G) in non-HAS and one (Signature H) in TCGA-LIHC (Figure 2E). Signature A and D were both highly similar to COSMIC mutational signature 1 (cosine similarity: 0.911 and 0.929, respectively), which was initiated by spontaneous deamination of 5-methylcytosine and correlated with age 31. It was well-known that aging contributed to tumorigenesis and development of gastric cancer 32. Signature B identified in HAS was similar to Signature 2 (cosine similarity = 0.800), which was attributed to activity of the AID/APOBEC family of cytidine deaminases. DNA mutation of AID/APOBEC cytidine deaminases could lead to genomic instability in cancers 33. Signature C was similar to Signature 19 (cosine similarity = 0.801), but the aetiology of Signature 19 remains unknown. Apart from Signature D, Signature E, F and G were also identified in non-HAS but absent in HAS, which were similar to Signature 6, 17, and 29, respectively. Signature 6 was associated with defective DNA mismatch repair. As expected, two MSI cases was identified in non-HAS, whereas none was found in HAS (Table S11). Besides, previous studies have reported that Signature 17 is commonly observed in gastrointestinal cancers 34, 35; however, the aetiology is still unclear. In addition, Signature 29 was linked with tobacco chewing habit, a risk factor for gastric cancer 31. As for TCGA-LIHC, Signature H was the predominant mutational signature, which was highly similar to Signature 22 (cosine similarity = 0.929). Signature 22 was recognized as the hallmark of exposure to aristolochic acid and often identified in urothelial carcinoma 36 and liver cancer 37.

### 3.4 Identification of key CNV regions in HAS compared with non-HAS and TCGA-LIHC

**Figure 3:**
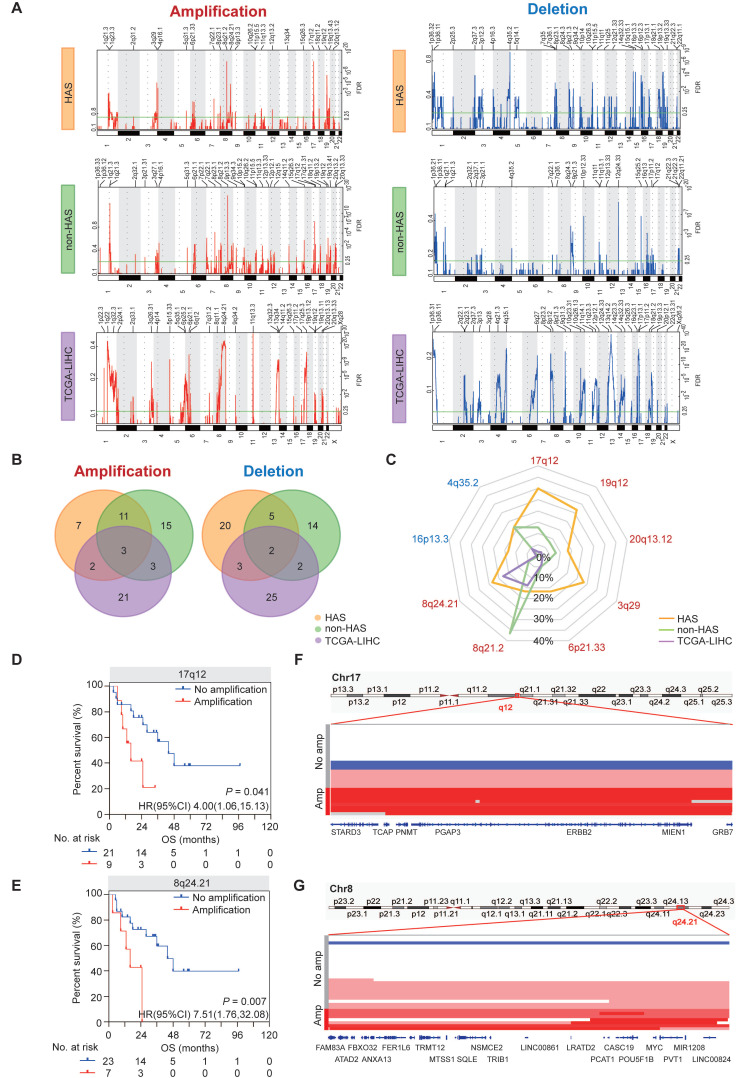
** Copy number variations in HAS, non-HAS, and TCGA-LIHC. (A)** Copy number profiles identified using GISTIC2.0 in HAS, non-HAS, and TCGA-LIHC, with amplification in red and deletion in blue. **(B)** Overlap of amplification (left panel) and deletion (right panel) between HAS, non-HAS, and TCGA-LIHC, respectively. **(C)** Comparison of significantly frequent CNVs in HAS, non-HAS, and TCGA-LIHC. **(D-E)** Association of 17q12 amplification **(D)** and 8q24.21 amplification **(E)** with OS in HAS, respectively. (**F-G**) Zooms in the significant amplification region in chromosome 17q12 **(F)** and 8q24.21 **(G)**. CNV, copy number variation.

The somatic CNV landscapes in HAS, non-HAS, and TCGA-LIHC were identified using GISTIC2.0 (Figure 3A). For HAS, most significant regions included amplification in17q12 (30.0%), 19q12 (26.7%), 8q24.21 (23.3%), and 3q29 (23.3%), and deletion in 16p13.3 (13.3%) and 4q35.2 (16.7%). For non-HAS, the most recurrent alterations were amplification in 8q21.2 (36.5%) and 1q21.3 (23.8%) and deletion in 12q24.33 (19.0%). For TCGA-LIHC, amplification in 11q13.3 and deletion in 13q14.2 occurred most frequently. Venn analyses revealed common and specific alterations between HAS, non-HAS, and TCGA-LIHC (Figure 3B). A total of 7 amplifications and 20 deletions were only found in HAS. Besides, amplification in 8q24.21 and 13q34, and deletion in 14q32.33, 2q37.3, and 17p13.1 were detected both in HAS and TCGA-LIHC but were not detected in non-HAS. Given the frequent CNVs (>10%) in HAS, we further compared these events between HAS, non-HAS, and TCGA-LIHC, including amplification in 17q12, 19q12, 20q13.12, 3q29, 6p21.33, 8q21.2, and 8q24.21, and deletion in 4q35.2 and 16p13.3 (Figure 3C and Table S12). Interestingly, 17q12 amplification was detected in HAS (30.0%) and non-HAS (12.7%) but was nearly absent in TCGA-LIHC (2.0%). 17q12 amplification occurred more frequently in HAS than non-HAS (*P* = 0.044). Remarkably, 8q24.21 amplification was common in HAS (23.3%) and TCGA-LIHC (17.7%) but rare in non-HAS (4.8%). Furthermore, Kaplan-Meier analysis identified poor prognosis of patients with 17q12 (HR = 4.00, 95%CI = [1.06, 15.13], *P* = 0.041; Figure 3D) and 8q24.21 amplification (HR = 7.51, 95%CI = [1.76, 32.08], *P* = 0.007; Figure 3E) rather than other regions (Figure S8). Numerous genes located in 17q12 region have been implicated in diverse cancers (Figure 3F), such as *ERBB2* for gastric cancer 38 and breast cancer 39, *CDK12* for prostate cancer 40, and *STARD3* for breast cancer 41. *MYC*, a well-known oncogene located in 8q24.21, was also frequently amplified in HAS (Figure 3G). Besides, 8q24.21 contained a cluster of genes for miRNA and lncRNA which were identified as key regulators in tumorigenesis and progression (Figure 3G), such as *PVT1* 42 and *PCAT1* 43.

### 3.5 Recurrent somatic genetic alterations of HAS were enriched in RTK/RAS/PI(3)K pathway, cell cycle, and signaling pathways regulating pluripotency of stem cells

**Figure 4:**
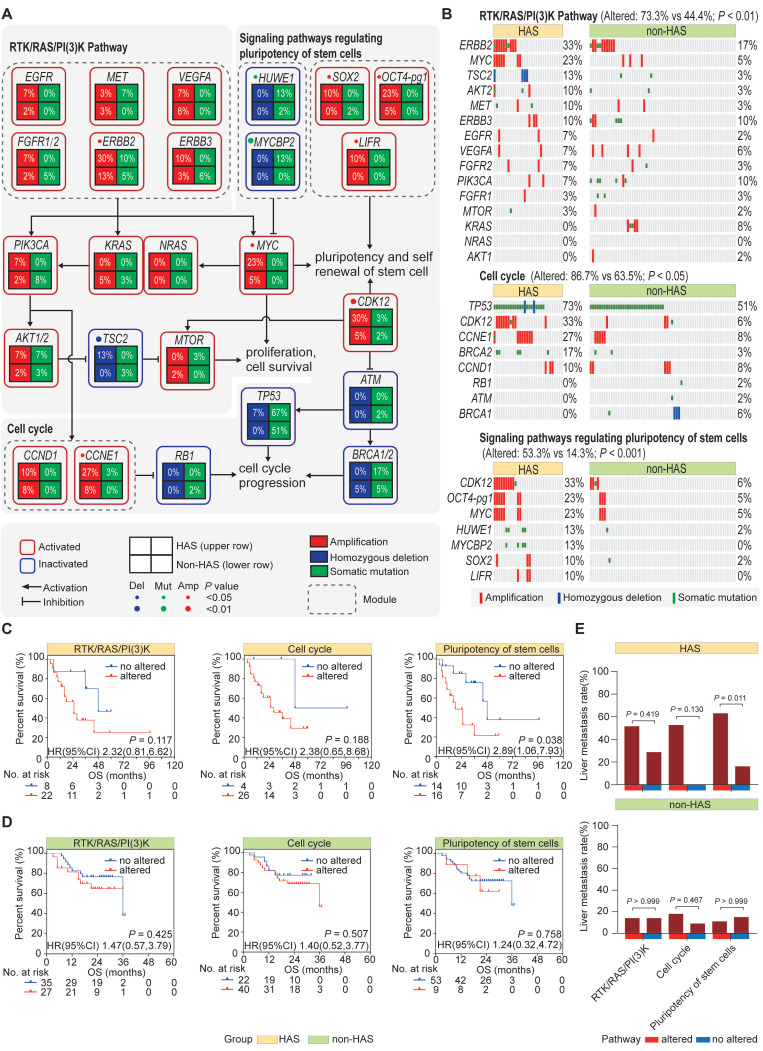
** Pathway enrichment analysis. (A)** Schematics of frequently altered pathways in HAS compared with non-HAS. Three key pathways including RTK/RAS/PI(3)K pathway, cell cycle pathway, and signaling pathways regulating pluripotency of stem cells were identified. Upper and lower row represents HAS and non-HAS in a square grid, respectively. The percentage represents the alteration frequency. **(B)** Comparison of genomic alterations involved in the enriched pathways between HAS and non-HAS.** (C-D)** Association of RTK/RAS/PI(3)K pathway, cell cycle pathway, and signaling pathways regulating pluripotency of stem cells with overall survival in HAS **(C)** and non-HAS **(D)**, respectively. **(E)** Association of RTK/RAS/PI(3)K pathway, cell cycle pathway, and signaling pathways regulating pluripotency of stem cells with liver metastasis in HAS and non-HAS.

Given the repertoire of somatic genetic alterations detected in HAS and non-HAS, we sought to identify signaling pathways targeted by differential somatic genomic alterations (SNVs and CNVs). KEGG pathway analysis revealed that different genomic alterations were enriched in RTK/RAS/PI(3)K pathway (*P*_adj_ = 0.002, Figure S9), cell cycle (*P*_adj_ = 0.047, Figure S9), and signaling pathways regulating pluripotency of stem cells (*P*_adj_ = 0.013, Figure S9). As displayed in Figure 4A-B, the most frequently altered gene in RTK/RAS/PI(3)K pathway was *ERBB2* alteration (33% vs 17%, *P* = 0.087), followed by *MYC* amplification (23% vs 5%, *P* = 0.012). In cell cycle pathway, several well-known cancer related genes were more frequently altered in HAS comparing to non-HAS, including *TP53* (73% vs 51%, *P* = 0.040), *CDK12* (33% vs 6%, *P* = 0.001), *CCNE1* (27% vs 8%, *P* = 0.024), and *BRCA2* (17% vs 3%, *P* = 0.034). Interestingly, in addition to *CDK12* and *MYC*, other genes involved in the signaling pathways regulating pluripotency of stem cells were also recurrently affected in HAS, including *OCT4-pg1* (23% vs 5%, *P* = 0.012), *HUWE1* (13% vs 2%, *P* = 0.036), and *MYCBP2* (13% vs 0%, *P* = 0.009). Collectively, given the somatic genetic alterations affecting at least one gene in the pathway, HAS showed more genetic instability than non-HAS in RTK/RAS/PI(3)K pathway (73.3% vs 44.1%, *P* < 0.01; Figure 4B), cell cycle (86.7% vs 63.5%, *P* < 0.05; Figure 4B), and signaling pathways regulating pluripotency of stem cells (53.3% vs 14.3%, *P* < 0.001; Figure 4B). Furthermore, Kaplan-Meier analysis revealed that HAS patients with the altered gene in signaling pathways regulating pluripotency of stem cells harbored worse OS than those without alterations (HR = 2.89, 95%CI = [1.06, 7.93], *P* = 0.038; Figure 4C). Besides, for RTK/RAS/PI(3)K and cell cycle pathways, altered groups in HAS both tended to show more unfavorable OS than unaltered groups, although it was not significant (RTK/RAS/PI(3)K: HR = 2.32, 95%CI = [0.81, 6.62], *P* = 0.117; cell cycle: HR = 2.38, 95%CI = [0.65, 8.68], *P* = 0.188; Figure 4C). In contrast, in non-HAS patients, little OS difference was observed between the groups with and without alteration in these three pathways (Figure 4D). Furthermore, liver metastasis tended to occur more frequently in HAS patients with the altered gene of cell cycle pathway (46% vs 0%, *P* = 0.130; Figure 4E) and signaling pathways regulating pluripotency of stem cells (63% vs 14%, *P* = 0.011; Figure 4E) than those without alteration. However, the associations of these pathway alterations with liver metastasis were all not significant in non-HAS patients (all *P* > 0.05, Figure 4E).

### 3.6 HAS developed abundant multiclonal architecture associated with liver metastasis

**Figure 5:**
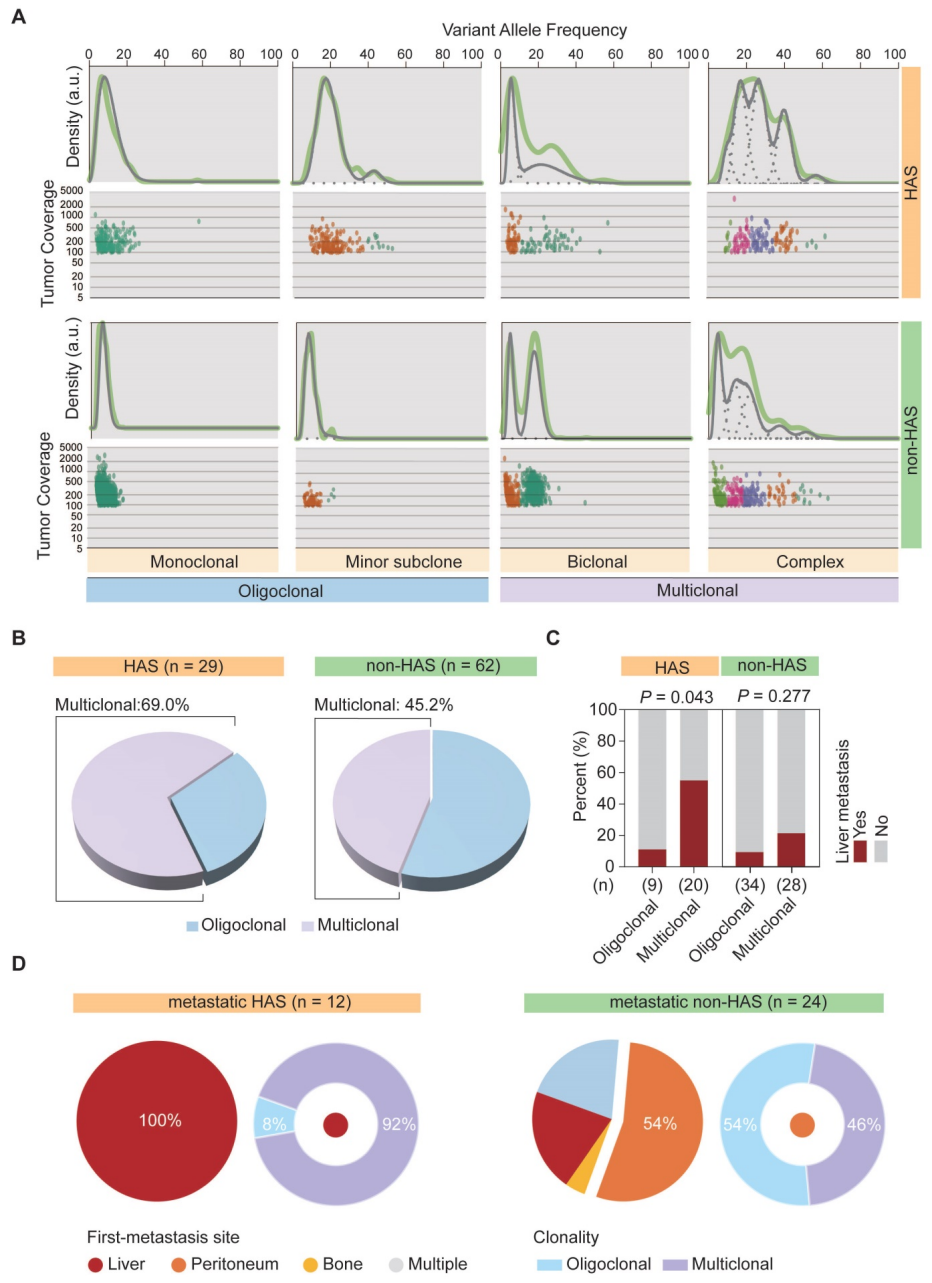
**The clonal architecture inferred in HAS and non-HAS. (A)** Patterns of inferred clonal architecture based on Kernel density plots of VAF and VAF plotted versus read depth. Monoclonal and minor subclonal are defined as the oligoclonal type, whereas biclonal and complex clonal are defined as the multiclonal type.** (B)** Comparison of clonal architecture between HAS and non-HAS. **(C)** Association of clonality with liver metastasis in HAS and non-HAS patients.** (D)** Association of clonality with metastatic pattern in HAS and non-HAS patients. The pie diagrams display the distribution of first-metastasis site in metastatic HAS and metastatic non-HAS patients. The circular diagrams display the distribution of clonal architecture in HAS and non-HAS patients with the most frequent metastasis site. VAF, variant allele frequency.

We previously observed that, most metastatic HAS patients only developed liver metastasis, whereas metastatic non-HAS patients developed multi-organ metastases. This dominantly different metastasis pattern might be explained using tumor clonality model. Therefore, SciClone analysis 27 was performed to reconstruct the clonal architecture in HAS and non-HAS samples. As depicted in Figure 5A, the raw clonality pattern was defined according to the clonal and subclonal architecture, including a single dominant clone (monoclonal), a dominant clone plus a minor subclone (minor subclone), two clones (biclonal), and finally, more than two clones (complex) 44. Monoclonal and minor subclones were defined as the oligoclonal type, whereas biclonal and complex were defined as the multiclonal type (Figure 5A). The clonality of each sample in HAS and non-HAS is listed in Table S13. Comparative analysis revealed that HAS harbored more abundant multiclonal type than non-HAS (69.0% vs 45.2%, *P* = 0.035; Figure 5B). In addition, HAS patients with multiclonal type developed higher rate of liver metastasis than those with oligoclonal type (55.0% vs 11.1%, *P* = 0.043; Figure 5C), whereas the association of clonal architecture with liver metastasis was not significant in non-HAS patients (21.4% vs 8.8%, *P* = 0.277; Figure 5C). Furthermore, we investigated the association of metastasis patterns with clonal architecture in HAS and non-HAS patients. As displayed in Figure 5D, 12 HAS and 24 non-HAS patients developed synchronous or metachronous metastasis. Interestingly, all HAS patients only developed liver metastasis (100%), whereas non-HAS patients developed multi-organ metastasis, mainly peritoneal (54%). Notably, most metastatic HAS were classified into multiclonal type (92%), whereas there was no dominant bias of the distribution of oligoclonal (54%) and multiclonal type (46%) in metastatic non-HAS (Figure 5D). Furthermore, these findings were validated by using other bioinformatic pipelines including PyClone and MOBSTER algorithms (Figure S10, Table S14).

### 3.7 Potential therapeutic targets for HAS

**Figure 6:**
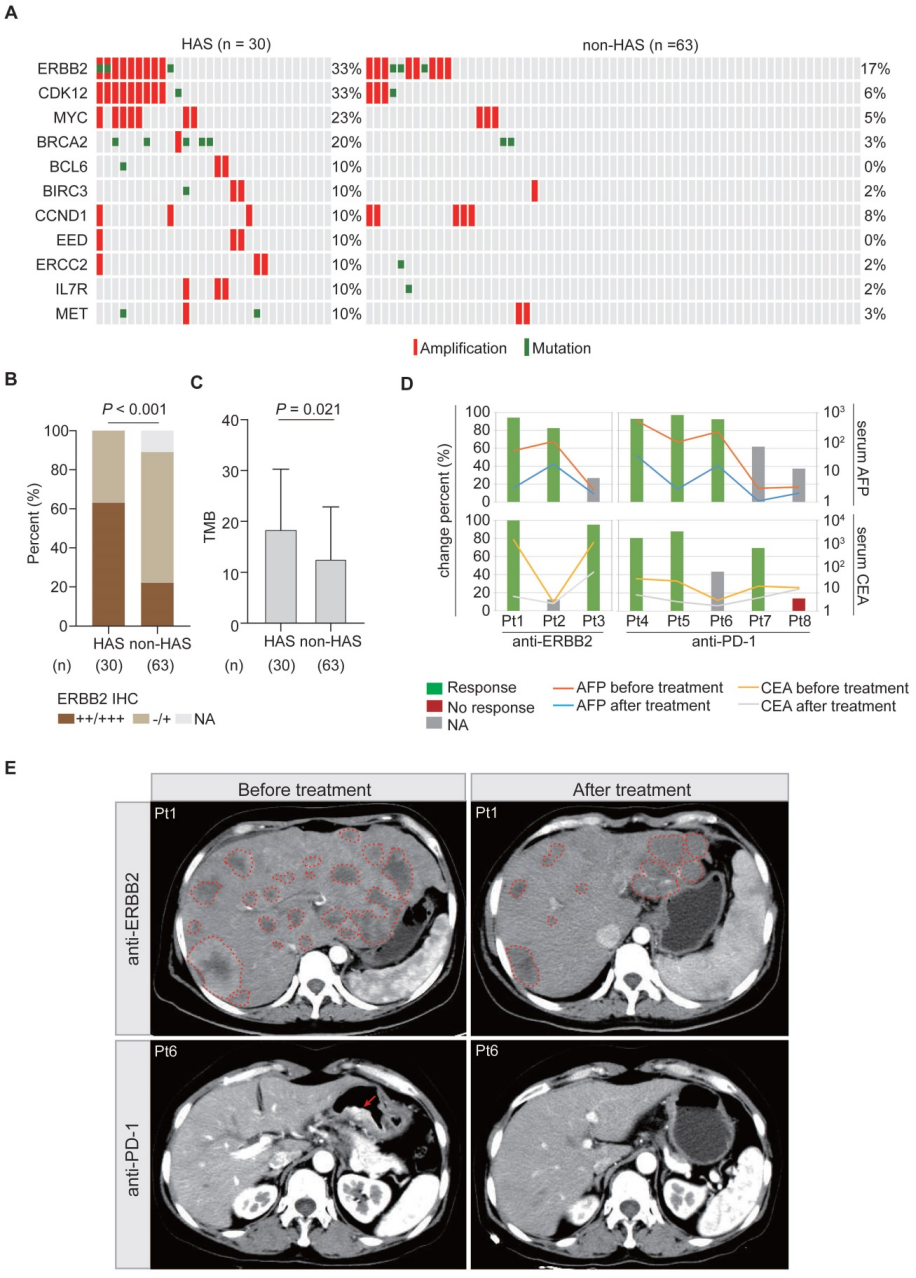
**Potential targets and therapy for HAS. (A)** Frequently altered genomic targets in HAS compared with non-HAS. **(B)** Comparison of ERBB2 expression between HAS and non-HAS. **(C)** Comparison of TMB between HAS and non-HAS.** (D)** Assessment of serum AFP and CEA levels in HAS patients before and after treatment. Three HAS patients received anti-ERBB2 therapy and five HAS patients received anti-PD-1 therapy. The line charts represent serum AFP/CEA level and histograms represent the CP of serum AFP/CEA level; details are shown as follows: CP (%) = (serum AFP or CEA levels before treatment - serum AFP or CEA levels after treatment) / serum AFP or CEA levels before treatment *100%. The values of 20 ng/mL and 5 ng/mL are defined as the serum AFP and CEA levels threshold, respectively. For each patient with elevated AFP/CEA level before treatment, 65% was defined as the cutoff for response to anti-ERBB2/anti-PD-1 therapy. Blue and red represent response and no response at the serum tumor biomarker levels, respectively. Grey means that this evaluation criterion is inapplicable for a patient with a normal AFP/CEA level before treatment. **(E)** Representative CT images of abdomen showing tumor lesions of two patients before and after treatment; one received anti-ERBB2 therapy and the other received anti-PD-1 therapy. The dotted line represents the metastatic liver lesion, and the arrow represents the primary gastric lesion. IHC, immunohistochemistry; AFP, alpha-fetoprotein; CEA, carcinoembryonic antigen; CP, change percent; Pt, patient.

Under the first-line systemic chemotherapy for CGC, the prognosis of HAS patients remains unfavorable 45. In this study, aiming to identify potential therapeutic targets for HAS, we matched the SNV and CNV profiles to the gene list of actionable genomic alterations obtained from OncoKB database. A total of 11 potential targets (≥ 10%) were identified in HAS and matched in non-HAS. Encouragingly, patients with ERBB2 amplification, who might benefit from well-known anti-ERBB2 therapy, were enriched in HAS (Figure 6A). Besides, *ERBB2* and *CDK12* were frequently co-amplified in HAS, which was not observed in non-HAS (Figure 6A). *MYC* amplification (23% vs 5%, *P* = 0.012) and *BRCA2* alteration (20% vs 3%, *P* = 0.013) also occurred more frequently in HAS than non-HAS. The drugs targeting these gene alterations are listed in Table S15. As expected, IHC testing validated that HAS harbored more abundant ERBB2 expression than non-HAS (*P* < 0.001, Figure 6B). In addition, HAS harbored more tumor mutation burden (a genomic biomarker for immunotherapy) than non-HAS (mean TMB: 18.5/Mb vs 12.6/Mb, *P* = 0.021; Figure 6C). Given the spectrum of potential targets and/or biomarkers for target therapy and immunotherapy, we evaluated the treatment efficiency for HAS patients receiving anti-ERBB2 (3 cases) or anti-PD-1 therapy (5 cases) by monitoring the change percent (CP) of serum AFP and CEA levels in HAS patients before and after treatment. Inspiringly, we observed that 100% (3/3) of patients showed dramatically decreased AFP and/or CEA levels (CP > 65%) after receiving anti-ERBB2 therapy (Figure 6D). In addition, serum AFP and/or CEA levels were markedly down-regulated (CP > 65%) in 80% (4/5) of patients treated with anti-PD-1 therapy (Figure 6D). Given the available CT images of two HAS patients during treatment period, we observed that the number of metastatic liver lesions decreased dominantly in a HAS patient after anti-ERBB2 therapy (Figure 6E). Besides, a representative CT image revealed a dramatic regression of the primary gastric lesion in another HAS patient after anti-PD-1 therapy (Figure 6E).

### 3.8 Comparison of the clinicogenomic features of HAS between this study and previous study

Recently, Liu et al. have reported the clinicogenomic features of HAS patients 14. Therefore, we conducted comparative analysis for our study and Liu et al.'s study. Firstly, we observed significant differences in clinicopathological characteristics of HAS patients between our study and Liu et al.'s study. As shown in Table S16, HAS patients were at a more advanced TNM stage in ZJU-WES cohort (n = 30) than those in Liu et al. cohort (*P* = 0.001). The proportion of metastatic patients was significantly higher in ZJU-WES cohort than Liu et al. cohort (M stage: 30.0% vs 1.8%, *P* < 0.001). Furthermore, the serum AFP level of HAS patients was significantly higher in the ZJU-WES cohort than Liu et al. cohort (median: 195.9 ng/mL vs 20.9 ng/mL, *P* = 0.005). Similar findings were also validated in the comparison between the ZJU-overall cohort (n = 90) and Liu et al. cohort (Table S17).

Therefore, it was nonnegligible that HAS patients were mainly at the advanced stage in our study, whereas those were mainly at the early stage in Liu et al.'s study. Currently, liver metastasis has been recognized as a hallmark event in advanced-stage HAS patients; however, the clinical risk factors and molecular basis associated with liver metastasis remain largely unknown at present. Limited by the non-metastatic cohort, Liu et al.'s study has not responded to this tricky question. On the contrary, our study provided a relatively ideal platform to explore the clinicogenomic landscape underlying liver metastasis and the poor prognosis of HAS patients. From the genomic perspective, *TP53* was identified as the only SMG for HAS in our study as well as in Liu et al.'s study 14. Although *TP53* mutation occurred in multiple cancer types, recent studies have recognized the genetic background of frequent *TP53* mutations as one of the genomic hallmarks in HAS^13^, ^46^. This genetic event might contribute to the production of AFP 47. Subsequently, we compared the frequently mutated genes (>10%) of HAS between our study and Liu et al.'s study. As shown in Figure S11, nearly 70% of genes from Liu et al.'s study was also observed in our study, such as *TP53*, *MUC16*, *ABCA13*, *FLG*, *ZFHX4*, etc. More importantly, our study demonstrated a more frequent mutation spectrum, such as *PCLO*, *MYCBP2*, *HUWE1*, etc. Mutations in these genes correlated with liver metastasis or poor prognosis in HAS. At the level of copy number alterations, several factors regulating stem cell differentiation were covered, such as *MYC* in 8q24.21 and *FAT1* in 4q35.2, consistent with Liu et al.'s findings. However, 17q12 amplification, the most frequent CNV in our cohort, was absent in Liu et al.'s study. This region contained a series of well-known genes involved in cancer development, such as *ERBB2*, *CDK12*, and *STARD3*. From the signaling pathway perspective, our study and Liu et al.'s study both uncovered the vital role of stem cell property in HAS. Liu et al. highlighted that high stemness activity play a key promotor for tumorigenesis in HAS using transcriptome sequencing analysis. Interestingly, our study demonstrated that HAS harbored frequent alterations in signaling pathways regulating stem cell pluripotency from the genomic level. At the level of clonal architecture, multiple bioinformatic pipelines including SciClone, PyClone and MOBSTER demonstrated that HAS developed abundant multiclonal architecture, which was correlated with liver metastasis. On the contrary, Liu et al. found that all HAS cases harbored a group of monoclonal mutations. Currently, it is believed that multiclonal seeding serves as one of the key biological mechanisms underlying cancer metastasis 48, 49. We considered that the baseline bias of metastasis stage between two studies was one of the leading causes for these different findings; Liu et al.'s study enrolled a non-metastatic HAS population (98.2%), whereas our study contained a relatively higher proportion of metastatic HAS patients (34.4%).

## 4. Discussion

This study depicted a clinicogenomic landscape of HAS based on large-scale clinical cohorts and multidimensional genomic data, providing valuable insights for a better understanding of molecular mechanisms underlying liver metastasis and unfavorable prognosis in HAS. By comparing clinical and genomic features between HAS, non-HAS, and TCGA-LIHC, this study highlights the following findings: (1) HAS harbored worse overall survival and developed a higher propensity of liver metastasis than non-HAS; (2) HAS presented a genomic landscape associated with the unfavorable clinical features, containing more frequent somatic mutations and widespread copy number variations than non-HAS and TCGA-LIHC, such as frequent *PCLO* mutation, and recurrent amplification in 17q12 and 8q24.21; (3) key signaling pathways affected by frequent genomic alterations might contribute to poor prognosis and liver metastasis in HAS, capital among these being the signaling pathways regulating pluripotency of stem cells; (4) HAS developed abundant multiclonal architecture associated with liver metastasis; and (5) a considerable proportion of HAS patients might benefit from anti-ERBB2 or anti-PD-1 therapy.

Due to the extremely low incidence, lack of uniform diagnostic criteria for HAS, and small sample size, little evidence for systematically summarizing the clinical features of HAS have been established to date. Most studies reported many HAS cases with liver metastasis and poor prognosis, and most of them were case reports or case series 5, 50, 51. Interestingly, Zhou et al. found that the prognosis of HAS patients might not be as unsatisfactory as previously believed 12. Similar findings were also observed by Osada et al. 11. In this study, we constructed the largest clinical cohort from our institution and performed integrative analysis to elucidate the key clinical features of HAS. Our study demonstrated that HAS patients harbored significantly worse OS and developed a higher rate of liver metastasis than non-HAS patients. Additionally, these findings were confirmed in SEER cohort and subsequent meta-analysis. Nevertheless, the meta-analysis revealed undeniable heterogeneity between studies, which might be partly explained by different diagnostic criteria for HAS. Three studies identified HAS based on histological hepatoid differentiation and AFP production 2, 3, 12, whereas the others, including our study, only used the morphology as the diagnostic criteria according to WHO classification 11, 15, 17. The subgroup analysis suggested that the difference in OS was non-negligible between AFP-producing HAS and common HAS (Table S4). In addition, despite several HAS cases with a metastatic lesion in brain 52, lung 10, or peritoneum 53, as previously reported, liver metastasis was considered the most frequent event in HAS patients. Liu et al. 2 and Dai et al. 17 reported liver metastasis in 75.6% (34/45) and 53.3% (8/15) of HAS patients, respectively. In this study, 41.1% (37/90) of HAS patients developed synchronous or metachronous liver metastasis. Remarkably, we observed that most metastatic HAS only developed liver metastasis (97.3%), whereas metastatic non-HAS developed multi-organ metastases, including peritoneum (35.3%), liver (34.3%), and ovary (3.9%). Overall, we speculated that the frequent occurrence of liver metastasis, as a hallmark event in advanced-stage HAS patients, eventually contributed to unfavorable prognosis. To this extent, it is crucial to illustrate the mechanism underlying liver metastasis and poor prognosis of HAS.

From a clinicopathological perspective, most HAS patients exhibited a dramatic elevation in serum AFP level 54, 55, which was considered a remarkable feature contributing to aggressive behavior and poor prognosis 53. Ye et al. reported a marked elevation in serum AFP level in two HAS cases with lymph nodes and/or liver metastases (5845 ng/mL and > 50000 ng/mL, respectively) 56. Wang et al. observed that serum AFP level of ≥ 500 ng/mL was significantly associated with worse OS in HAS patients 13. In this study, serum AFP level was higher in HAS than non-HAS patients (76.7% vs 4.8%, Table 1). Notably, serum AFP level was identified as an independent risk factor for OS and liver metastasis of HAS patients (Figure S12). Furthermore, in patients undergoing radical surgery, postoperative serum AFP level was significantly lower than the preoperative level (*P* < 0.001, Figure S13). A previous study reported a HAS case that developed distant metastasis following surgery, along with a dramatic elevation in serum AFP level 56. Therefore, we considered that AFP was a key promoter associated with liver metastasis and poor prognosis in HAS patients. In addition, a previous report identified CEA, another tumor marker, as an independent risk factor for OS 57. Similar findings were also observed in this study (Figure S12). Overall, monitoring serum AFP and CEA levels of HAS patients is recommended in clinical practice for treatment evaluation and prognosis prediction. Besides, vascular invasion occurred more frequently in HAS than non-HAS patients in ZJU cohort (Figure S14A), as well as validated in the meta-analysis (Figure S14B). However, the difference in lymph node metastasis was not significant between HAS and non-HAS patients (Figure S14C-D). Zeng et al. also observed that 69.6% (199/286) of HAS tumors developed vascular invasion 5. Lin et al. reported that the presence of isolated portal vein tumor thrombosis was considered a risk factor for liver metastasis in HAS patients 7. Therefore, we considered that vascular invasion might be a crucial mechanism of liver metastasis in HAS.

This study characterized the genomic landscape and clinical correlations in HAS and conducted a comparative analysis between HAS, non-HAS, and TCGA-LIHC. Mutational analysis revealed that HAS displayed a frequent mutational spectrum containing a list of cancer-related genes compared with non-HAS and TCGA-LIHC, such as *TP53*, *PCLO*, and *BRCA2*. The enrichment of *TP53* mutation was considered as a key genomic feature in previous studies. Wang et al. 13 and Akiyama et al. 58 independently identified *TP53* as a frequently mutated gene in HAS. Furthermore, Liu et al. found that *TP53* mutations were shared between hepatoid adenocarcinoma and tubular adenocarcinoma components, implying a pivotal role in the phenotypic transition of HAS 14. *PCLO* mutation commonly occurred in diverse cancers, such as esophageal cancer 59, liver cancer 60, and colon cancer 61. Zhang et al. demonstrated that deregulation of the presynaptic cytomatrix protein Piccolo, encoded by *PCLO*, contributed to tumor aggressiveness and poor prognosis in esophageal cancer 59. In this study, we observed that *PCLO* mutation was also significantly associated with poor prognosis in HAS patients (Figure 2D). *BRCA2* mutation occurred frequently in breast cancer 62, ovarian cancer 63, and prostate cancer 64. Functionally, *BRCA2* mutation affected the homologous recombination repair mechanism, ultimately resulting in genomic instability in tumor cells 65. Notably, *BRCA2* mutation has been validated as a genetic target for PARP inhibitors in breast cancer 62, providing treatment hopes for HAS patients with a *BRCA2* mutation. At the CNV level, we identified amplification in 17q12 and 8q24.21 as the key genomic events associated with OS in HAS. On chromosome 17q12, a well-known oncogene *ERBB2* (*HER2*) coexisted with other cancer-related genes, such as *CDK12* 40, *STARD3* 66, and *GRB7* 67. We considered that a high level of amplification in these genes might contribute to aggressive behavior and poor prognosis of HAS. In addition, amplification in 8q24.21 frequently occurred in HAS and TCGA-LIHC but rarely in non-HAS (Figure 3C). As a famous oncogene at 8q24.21 region, *MYC* was identified as one of the core genes regulating stem cell-like properties of cancer cells 68. A recent study illustrated that high-efficiency c-Myc enhanced the conversion from mesenchymal stem cells to hepatoblast-like cells 69. In this study, we found that *MYC* and its regulators (*HUWE1* and *MYCBP2*) were enriched in the signaling pathways regulating pluripotency of stem cells (Figure 4A-B). Furthermore, frequent alterations in the signaling pathways regulating pluripotency of stem cells were significantly associated with poor prognosis (Figure 4C) and liver metastasis (Figure 4E) in HAS. Therefore, we suppose that overactivation of *MYC* function axis might initiate hepatoid differentiation in HAS by affecting the pluripotency of stem cells, and potentially contributed to miserable prognosis of HAS. Interestingly, Liu et al. 14 found that HAS had high stemness using bulk RNA-seq and scRNA-seq analyses, consistent with our findings based on the genomic analysis. Overall, the pluripotency of stem cells played a crucial role in hepatoid differentiation, liver metastasis and unfavorable prognosis in HAS, deserving further validation in vitro/in vivo experiments.

The clonal origin of HAS remains a debatable topic at present. Akiyama et al. found a same pattern of chromosome X inactivation, *TP53* mutation, and loss of heterozygosity in hepatoid and tubular adenocarcinomatous components and inferred that hepatoid differentiation was of identical origin to coexisting tubular differentiation in HAS 58. Liu et al. supported that hepatoid and tubular differentiation originated monoclonal by clonal analysis 14. However, Tsuruta et al demonstrated that HAS was a genetically heterogeneous group containing different genomic subtypes 70. In this study, SciClone analysis revealed that HAS harbored more abundant multiclonal architectures than non-HAS, representing a high level of intratumor heterogeneity in HAS. Furthermore, HAS with a multiclonal architecture developed a high rate of liver metastasis. From a mechanism perspective, we boldly speculated that genetically heterogeneous HAS cells in the primary site are selected for cluster formation, disseminated into vascular channels, and developed liver metastasis. Overall, the clonal origin of HAS remains controversial due to the complexity and heterogeneity of differentiation patterns, necessitating additional investigation and validation in large-scale cohorts.

Under platin-based chemotherapy, the first-line systemic regimen for metastatic GC, the prognosis of HAS patients remains unfavorable 45. A recent study revealed that platinum drug resistance-related genes were upregulated in HAS, implying that traditional chemotherapy was an unsatisfactory treatment option for HAS 14. Alternative strategies such as target therapy and immunotherapy are potentially efficient; however, limited molecular evidence supports their clinical application in HAS. Encouragingly, we discovered that HAS tumors harbored abundant ERBB2 expression and high TMB, which are biomarkers for anti-ERBB2 and anti-PD-1 therapies, respectively. Furthermore, we observed that 100% (3/3) and 80% (4/5) of patients showed dramatically decreased AFP and/or CEA levels after receiving anti-ERBB2 therapy and anti-PD-1 therapy, respectively. Interestingly, Fakhruddin et al. also reported that a HAS case with ERBB2 amplification achieved a partial response after anti-ERBB2 therapy 71. Li et al. observed that 85.7% (6/7) of AFP-producing GC/HAS patients had an evident clinical response to immunotherapy and recommended this optional treatment for the lethal disease 45. Mechanically, Liu et al. used scRNA-seq for a HAS case and revealed that high expression of activation markers GZMA and IFNG, as well as immune checkpoints PDCD1 and CTLA4, provided favorable conditions for immunotherapy 14. Besides, high TMB level, along with potential neoantigen production, might activate T-cells effector and improve response to immunotherapy 72. In summary, these findings supported that HAS patients might potentially benefit from anti-ERBB2 or anti-PD-1 therapy, warranting additional further investigation in clinical trials.

There are several limitations in this study. First, although this study comprised the largest clinical cohort to date, it remains difficult to timely obtain HAS samples due to extremely low incidence and rare surgical resection for this aggressive disease. Hence, genomic analysis results are limited by the small sample size. Second, liver metastasis was identified as a hallmark event for metastatic HAS patients. This organ-specific metastasis pattern with high occurrence observed in this study requires additional functional analysis using preclinical models to elucidate its molecular mechanism. In addition, the single-center retrospective design might introduce potential selection bias. Besides, the efficiency evaluation for anti-ERBB2 and anti-PD-1 therapies was mainly based on the limited number of HAS patients, requiring further investigation in larger clinical cohorts and validation in prospective clinical trials.

## 5. Conclusions

This study systematically demonstrated a high risk of liver metastasis and poor prognosis in HAS, elucidated the clinicogenomic landscape underlying these unfavorable clinical characteristics, and provided novel therapeutic insights into HAS. These findings lay the foundations for developing more precise diagnostic and therapeutic strategies for this rare but lethal disease.

## Supplementary Material

Supplementary figures and tables.

## Figures and Tables

**Table 1:** Clinicopathological characteristics of HAS and stage-matched non-HAS patients

Characteristic	HAS (N = 90)	Stage-matched non-HAS (N = 270)	*P*
**Age -years**			0.420
≤ 60	33(36.7)	112(41.5)	
> 60	57(63.3)	158(58.5)	
**Gender**			0.265
Female	24(26.7)	89(33.0)	
Male	66(73.3)	181(67.0)	
**T stage**			0.114
T1/2	22(24.4)	47(17.4)	
T3/4	48(53.3)	166(61.5)	
Unknown	20(22.2)	57(21.1)	
**N stage**			0.362
N0	19(21.1)	40(14.8)	
N1	12(13.3)	42(15.6)	
N2	19(21.1)	52(19.3)	
N3	20(22.2)	79(29.3)	
Unknown	20(22.2)	57(21.1)	
**M stage**			> 0.999
M0	59(65.6)	177(65.6)	
M1	31(34.4)	93(34.4)	
**AJCC stage**			> 0.999
I	9(10.0)	27(10.0)	
II	16(17.8)	48(17.8)	
III	34(37.8)	102(37.8)	
IV	31(34.4)	93(34.4)	
**Size -cm**			0.251
≤ 5.0	47(52.2)	161(59.6)	
> 5.0	23(25.6)	56(20.7)	
Unknown	20(22.2)	53(19.6)	
**Location**			0.088
Antrum	43(47.8)	146(54.1)	
Body	22(24.4)	77(28.5)	
Cardia	25(27.8)	46(17.0)	
Unknown	0(0)	1(0.4)	
**Vascular invasion**			< 0.001
Positive	44(48.9)	81(30.0)	
Negative	23(25.6)	124(45.9)	
Unknown	23(25.6)	65(24.1)	
**Serum AFP level -ng/mL**			< 0.001
≤ 20.0	21(23.3)	255(94.4)	
> 20.0	69(76.7)	13(4.8)	
Unknown	0(0)	2(0.7)	
**Serum CEA level -ng/mL**			0.009
≤ 5.0	47(52.2)	191(70.7)	
> 5.0	37(41.1)	77(28.5)	
Unknown	6(6.7)	2(0.7)	
**Serum CA199 level -U/mL**			0.341
≤ 37.0	69(76.7)	207(76.7)	
> 37.0	15(16.7)	61(22.6)	
Unknown	6(6.7)	2(0.7)	
**ERBB2 IHC status**			0.003
-/+	44(48.9)	158(58.5)	
++/+++	34(37.8)	54(20.0)	
Unknown	12(13.3)	58(21.5)	
**Surgery type**			0.062^d^
Distal gastrectomy	35(38.9)	143(53.0)	
Proximal gastrectomy	2(2.2)	3(1.1)	
Total gastrectomy	34(37.8)	75(27.8)	
No surgery	19(21.1)	49(18.1)	
**Chemotherapy^a^**			0.256
Received	67(74.4)	191(70.7)	
Not received	20(22.2)	79(29.3)	
Unknown	3(3.3)	0(0)	
**Target therapy^b^**			0.228^d^
Received	3(3.3)	8(3.0)	
Not received	84(93.3)	262(97.0)	
Unknown	3(3.3)	0(0)	
**Immunotherapy^c^**			< 0.001^d^
Received	6(6.7)	1(0.4)	
Not received	81(90.0)	269(99.6)	
Unknown	3(3.3)	0(0)	

^a^ Chemotherapy regimen was mainly based on the 5-fluorouracil (5-FU) plus platinum combination, including fluorouracil, leucovorin plus oxaliplatin (FOLFOX), oxaliplatin plus S-1 (SOX), and capecitabine plus oxaliplatin (XELOX). Other regimens included S-1, docetaxel, and paclitaxel plus S-1 (SPA). ^b^ Target therapy regimen included trastuzumab, bevacizumab, and apatinib. ^c^ Immunotherapy regimen included sintilimab and nivolumab. ^d^ Statistical analysis was conducted using Fisher's exact test, and other categorical data were using the chi-square test. The cases with unknown data were not included in the statistical analysis.

## Abbreviations

AFP): Alpha-fetoprotein
CA199): carbohydrate antigen 19-9
CEA): carcinoembryonic antigen
CGC): conventional gastric cancer
CNV): copy number variation
HAS): hepatoid adenocarcinoma of the stomach
HCC): hepatocellular carcinoma
non-HAS): non-hepatoid adenocarcinoma of the stomach
SEER): the Surveillance, Epidemiology, and End Results database
SNVs): single-nucleotide variants
TCGA-LIHC): hepatocellular carcinoma from The Cancer Genome Atlas
TMB): tumor mutation burden
VAFs): variant allele frequencies
WES): whole-exome sequencing
