# Supplementary material for: Integrative analysis reveals a clinicogenomic landscape associated with liver metastasis and poor prognosis in hepatoid adenocarcinoma of the stomach
Source: Int J Biol Sci. 2022 Aug 29;18(14):5554–74. doi: 10.7150/ijbs.71449 (PMC9461653; doi:10.7150/ijbs.71449)
Supplement: Supplementary file 1 — Supplementary figures and tables. [file ijbsv18p5554s1.pdf]

Supplementary Materials

1. Supplementary Figure

Figure S1. Comparison of overall survival between HAS and CGC using Kaplan-Meier analysis. HAS, hepatoid adenocarcinoma of the stomach; CGC, conventional gastric cancer; HR, hazard ratio; CI, confidence interval.

Figure S2. Forest plot showing COX analysis for overall survival in ZJU cohort. (A) Univariate COX analysis; **(B)** Multivariate COX analysis. Variables include age, gender, TNM stage, and pathological feature. The hazard ratios of each variable are presented and the horizontal lines indicate the 95% confidence intervals.

Figure S3. Comparison of prognosis and metastasis pattern between HAS and non-HAS in SEER cohort. (A) Overall survival; **(B)** Liver metastasis rate; **(C)** First-metastasis site. SEER, Surveillance, Epidemiology, and End Results database;

Figure S4. PRISMA flow diagram of the search process in the meta-analysis. [#]The data of HAS and non-HAS patients were collected from our institution (named as ZJU cohort).

Figure S5. Sensitivity analysis and publication bias analysis. (A-B) Sensitivity analysis for overall survival **(A)** and liver metastasis **(B)**. **(C-D)** Assessment of publication bias for overall survival using Begg's test **(C)** and Egger's test **(D)**. **(E-F)** Assessment of publication bias for liver metastasis using Begg's test **(E)** and Egger's test **(F)**.

Figure S6. Development of a Nomogram model for predicting liver metastasis in HAS patients. (A) Nomogram model based on results of multivariate logistic regression analysis in the training cohort. A total of six variables were enrolled in the model, including age, serum level of AFP, CA19-9, CA125, GLB, and ALP. **(B-C)** ROC analysis for evaluating the predictive value of the Nomogram model in the training cohort **(B)** and validation cohort **(C)**. **(D-E)** Calibration curve analysis for the Nomogram model in the training cohort **(D)** and validation cohort **(E)**.

Figure S7. Association of mutated genes with liver metastasis.

Figure S8. Association of significantly frequent CNVs with overall survival in HAS. (A) 19q12 amplification; **(B)** 3q29 amplification; **(C)** 6p21.33 amplification; **(D)** 20q13.12 amplification; **(E)** 8q21.2 amplification; **(F)** 4q35.2 deletion; **(G)** 16p13.3 deletion.

Figure S9. KEGG pathway enrichment analysis.

Figure S10. The clonal architecture inferred in HAS and non-HAS using PyClone and MOBSTER.

(A) Comparison of clonal architecture between HAS and non-HAS. **(B)** Association of clonality with liver metastasis in HAS and non-HAS patients. **(C)** Association of clonality with metastatic pattern in HAS and non-HAS patients. The pie diagrams display the distribution of first-metastasis site in metastatic HAS and metastatic non-HAS patients. The circular diagrams display the distribution of clonal architecture in HAS and non-HAS patients with the most frequent metastasis site.

Figure S11. Frequently mutated genes of HAS in Liu et al.'s study.

Figure S12. Forest plot showing COX analysis (A-B) for OS and logistic analysis (C-D) for liver metastasis in HAS. (A) Univariate COX analysis; **(B)** Multivariate COX analysis; **(C)** Univariate logistic analysis; **(D)** Multivariate logistic analysis. Variables include age, gender, serum AFP and CEA level, and ERBB2 IHC score. The hazard ratios or odds ratios of each variable are presented and the horizontal lines indicate the 95% confidence intervals.

Figure S13. Comparison between preoperative and postoperative level of serum tumor markers for HAS patients receiving radical surgery. (A) AFP; **(B)** CEA.

Figure S14. Comparison of clinicopathological features between HAS and non-HAS in ZJU cohort and Meta-analysis. (A-B) vascular invasion in ZJU-cohort **(A)** and meta-analysis **(B); (C-D)** lymph node metastasis in ZJU-cohort **(C)** and meta-analysis **(D)**.

2. Supplementary Table

Table S1. Comparison of clinicopathological characteristics between HAS and CGC patients

Table S2. Baseline characteristics of included studies in the meta-analysis

Table S3. Quality assessment of included studies using Newcastle-Ottawa scale

Table S4. Subgroup analysis for the comparison of OS between HAS and non-HAS patients

Table S5. Baseline characteristics of HAS patients in the training cohort and validation cohort

Table S6. Univariate analysis for factors associated with liver metastasis of HAS patients in the training cohort

Table S7. Multivariate Logistic regression analysis for factors associated with liver metastasis of HAS patients in the training cohort

Table S8. Sequencing depth of WES performing on the genomic DNA of tumor and normal sample in HAS and non-HAS

Table S9. Comparison of frequently mutated genes between HAS and non-HAS

Table S10. Comparison of frequently mutated genes between HAS and TCGA-LIHC

Table S11. Comparison of four TCGA subtypes between HAS and non-HAS

Table S12. Comparison of frequent and significant CNVs among HAS, non-HAS, and TCGA-LIHC

Table S13. The tumor clonality of each sample in HAS and non-HAS

Table S14. Clonal architecture of each sample identified using SciClone, PyClone, and MOBSTER

Table S15. Target therapies for gene alterations and relevant clinical trials or preclinical studies in cancer therapy

Table S16. Comparison of clinicopathological characteristics of HAS patients between ZJU-WES cohort and Liu et al. cohort

Table S17. Comparison of clinicopathological characteristics of HAS patients between ZJU-overall cohort and Liu et al. cohort

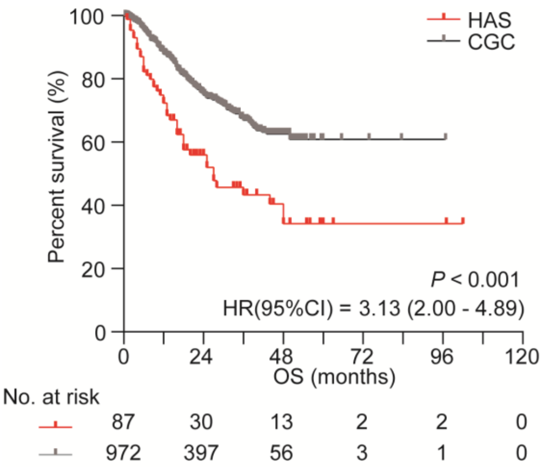

Figure S1. Comparison of overall survival between HAS and CGC using Kaplan-Meier analysis. HAS, hepatoid adenocarcinoma of the stomach; CGC, conventional gastric cancer; HR, hazard ratio; CI, confidence interval.

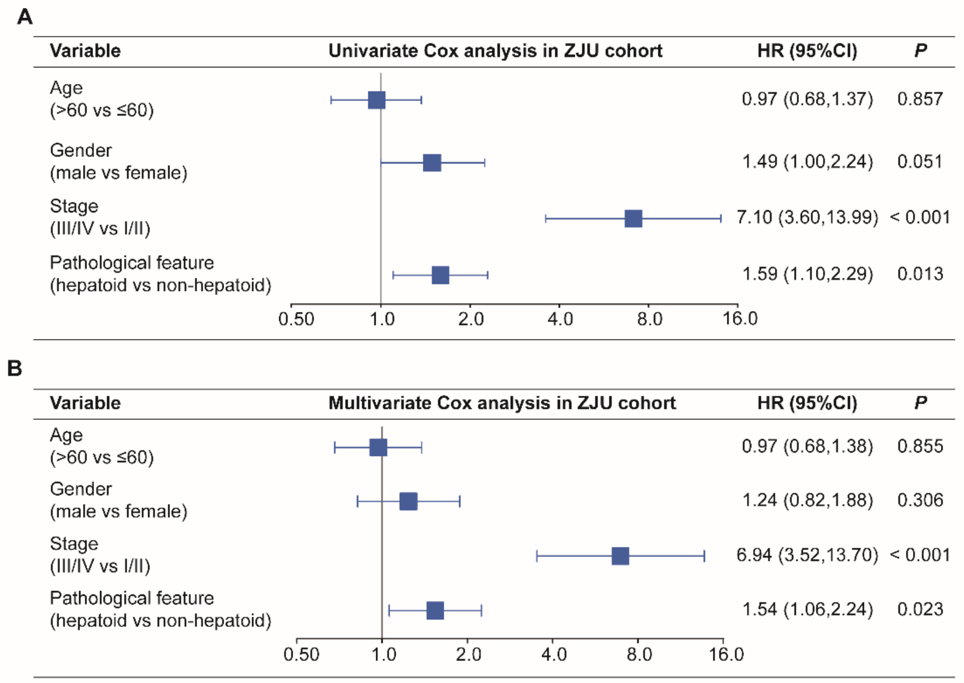

Figure S2. Forest plot showing COX analysis for overall survival in ZJU cohort. (A) Univariate COX analysis; **(B)** Multivariate COX analysis. Variables include age, gender, TNM stage, and pathological feature. The hazard ratios of each variable are presented and the horizontal lines indicate the 95% confidence intervals.

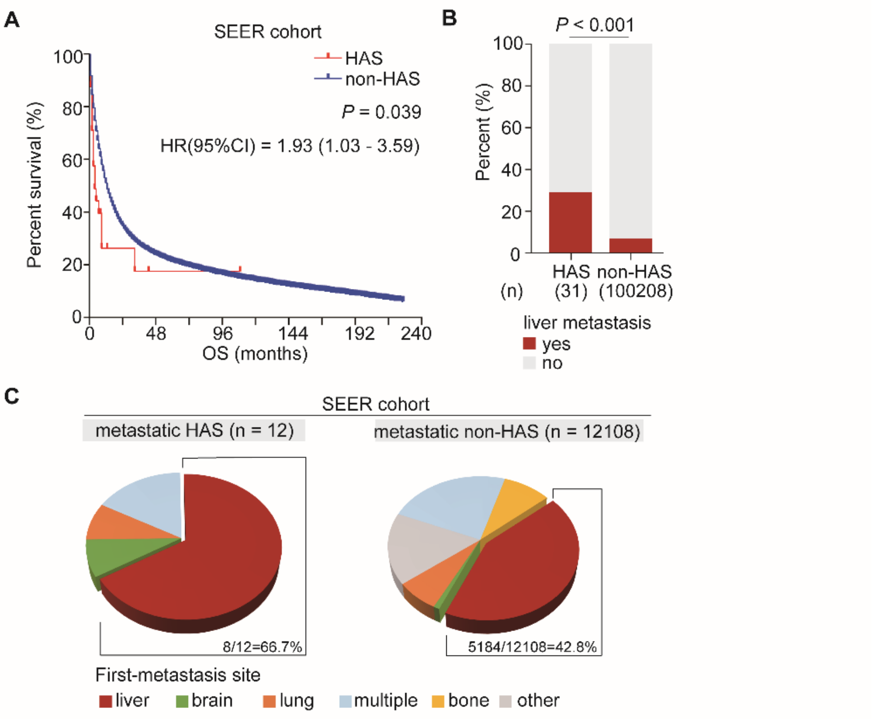

Figure S3. Comparison of prognosis and metastasis pattern between HAS and non-HAS in SEER cohort. (A) Overall survival; **(B)** Liver metastasis rate; **(C)** First-metastasis site. SEER, Surveillance, Epidemiology, and End Results database;

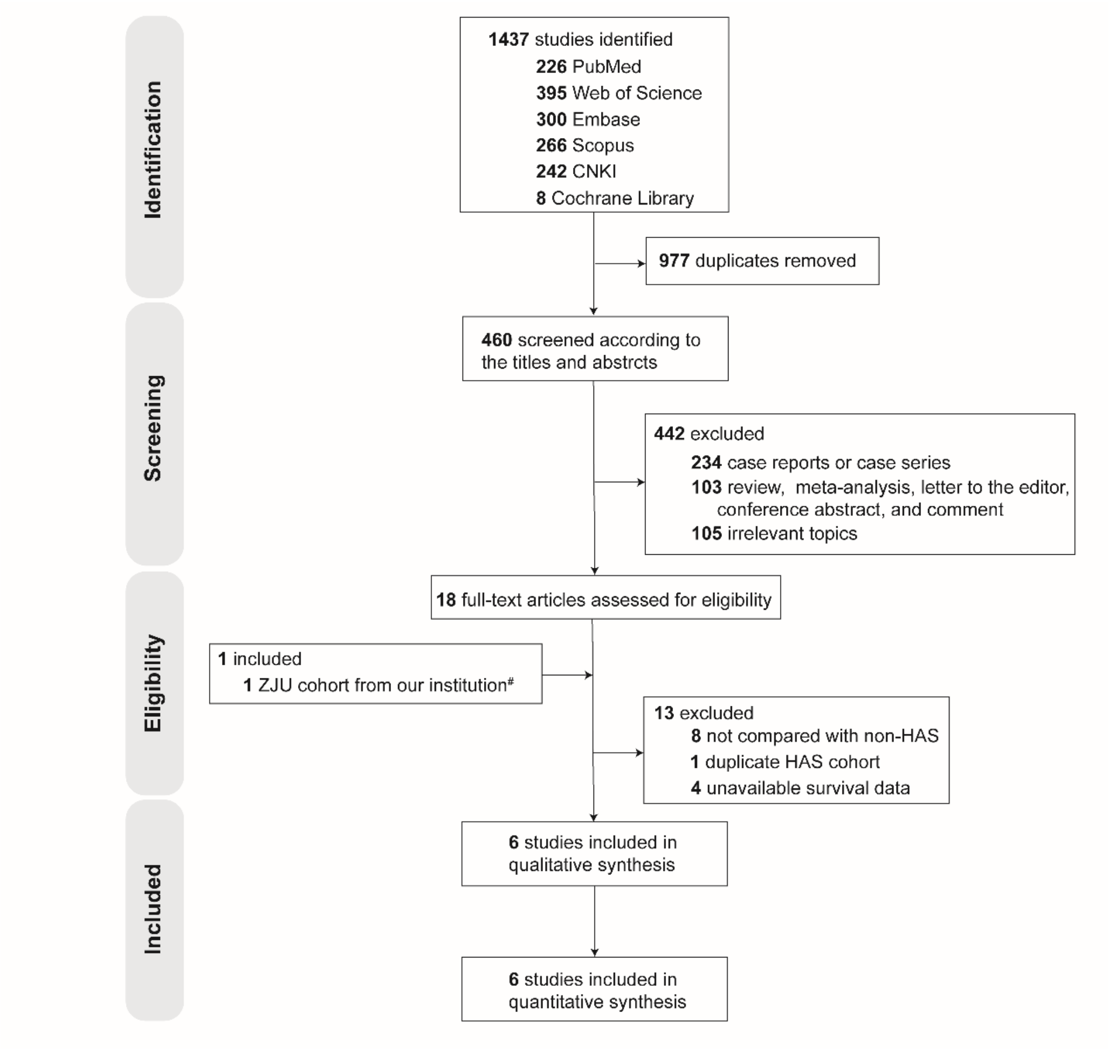

Figure S4. PRISMA flow diagram of the search process in the meta-analysis. [#]The data of HAS and non-HAS patients were collected from our institution (named as ZJU cohort).

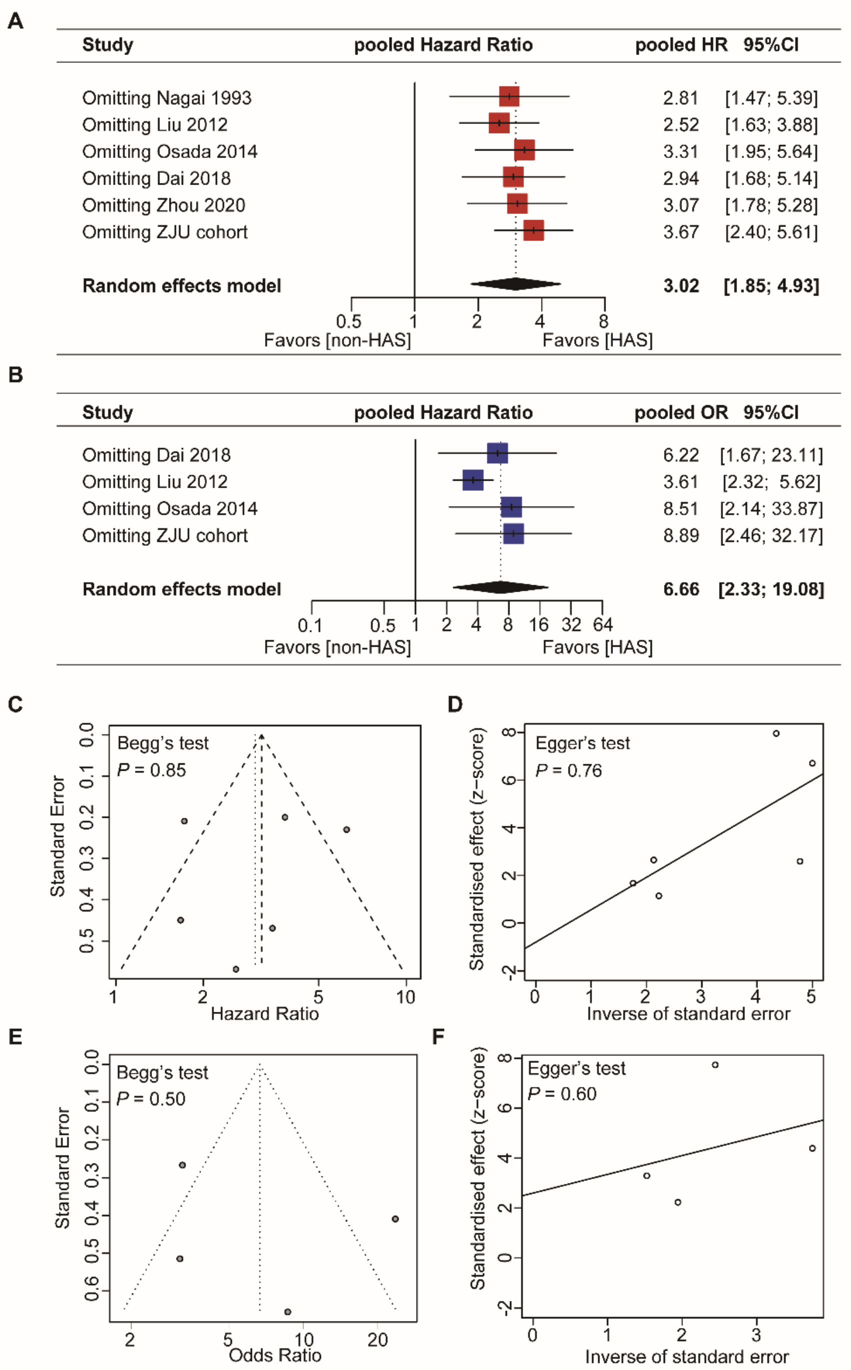

Figure S5. Sensitivity analysis and publication bias analysis. (A-B) Sensitivity analysis for overall survival **(A)** and liver metastasis **(B)**. **(C-D)** Assessment of publication bias for overall survival using Begg's test **(C)** and Egger's test **(D)**. **(E-F)** Assessment of publication bias for liver metastasis using Begg's test **(E)** and Egger's test **(F)**.

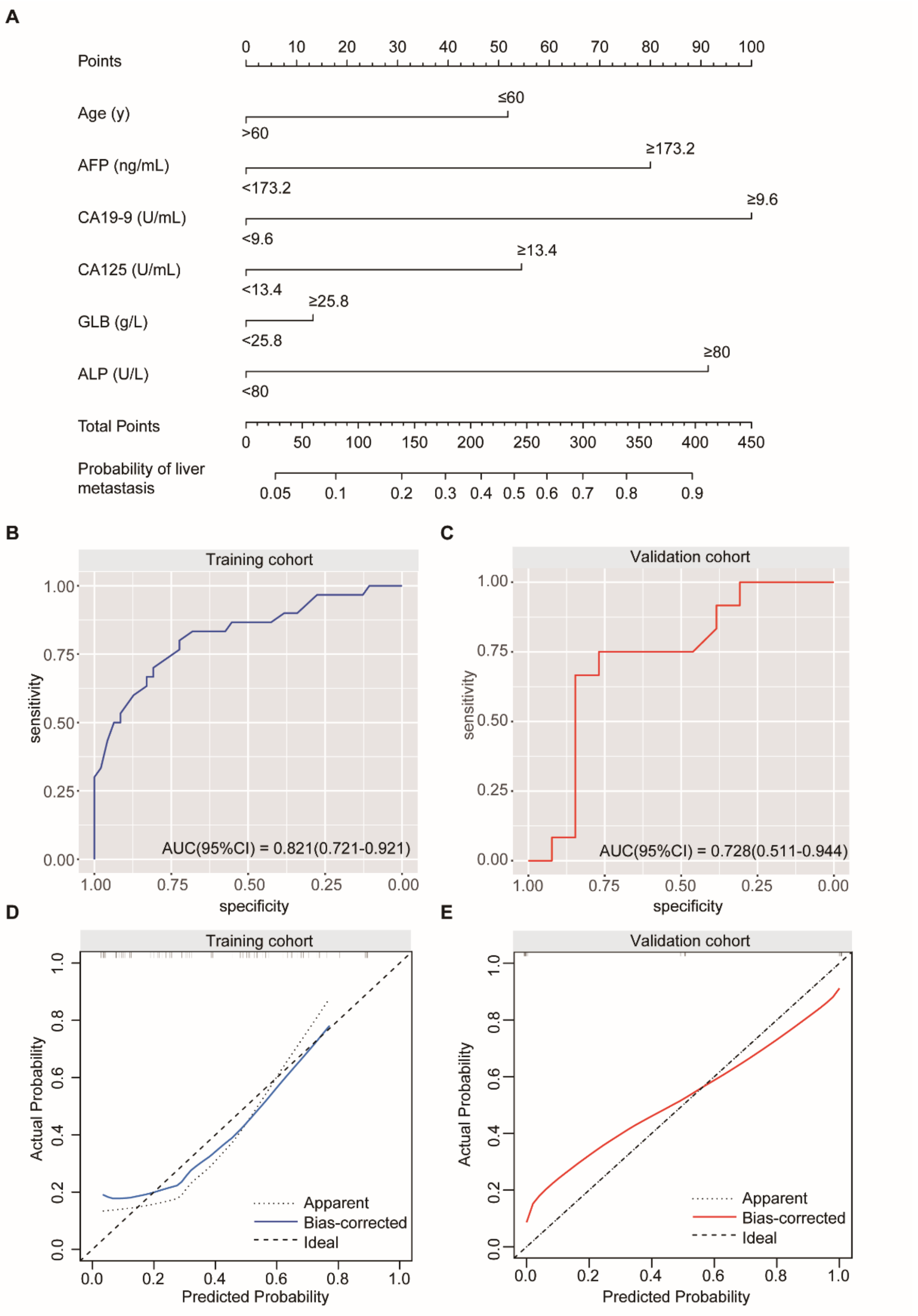

Figure S6. Development of a Nomogram model for predicting liver metastasis in HAS patients. (A) Nomogram model based on results of multivariate logistic regression analysis in the training cohort. A total of six variables were enrolled in the model, including age, serum level of AFP, CA19-9, CA125, GLB, and ALP. **(B-C)** ROC analysis for evaluating the predictive value of the Nomogram model in the training cohort **(B)** and validation cohort **(C)**. **(D-E)** Calibration curve analysis for the Nomogram model in the training cohort **(D)** and validation cohort **(E)**.

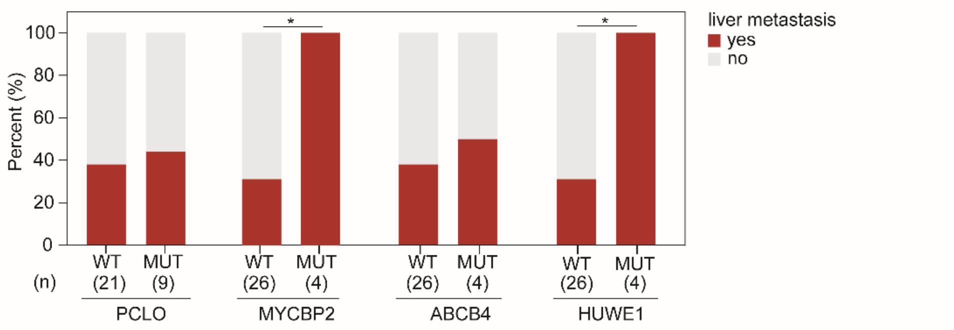

Figure S7. Association of mutated genes with liver metastasis.

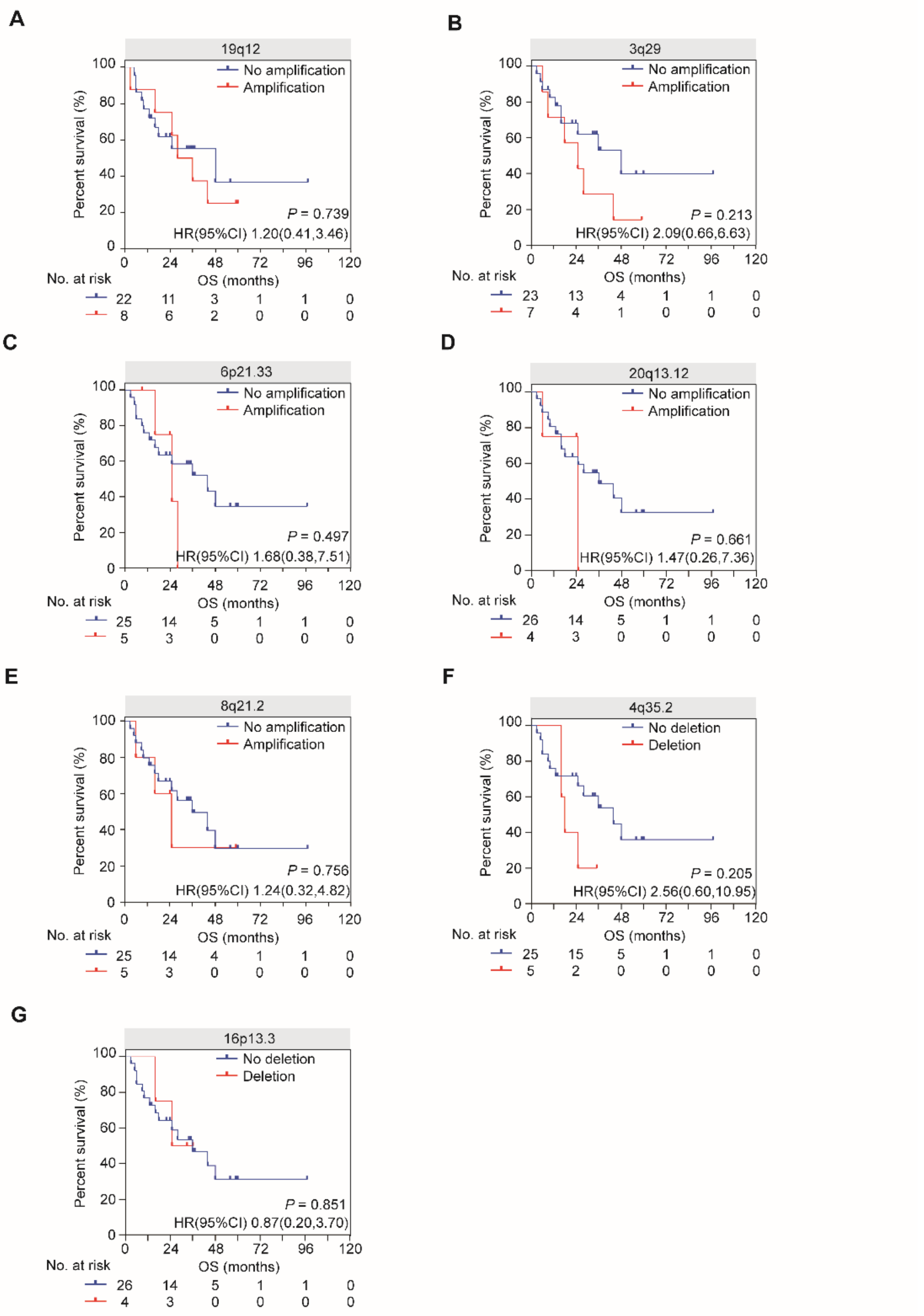

Figure S8. Association of significantly frequent CNVs with overall survival in HAS. (A) 19q12 amplification; **(B)** 3q29 amplification; **(C)** 6p21.33 amplification; **(D)** 20q13.12 amplification; **(E)** 8q21.2 amplification; **(F)** 4q35.2 deletion; **(G)** 16p13.3 deletion.

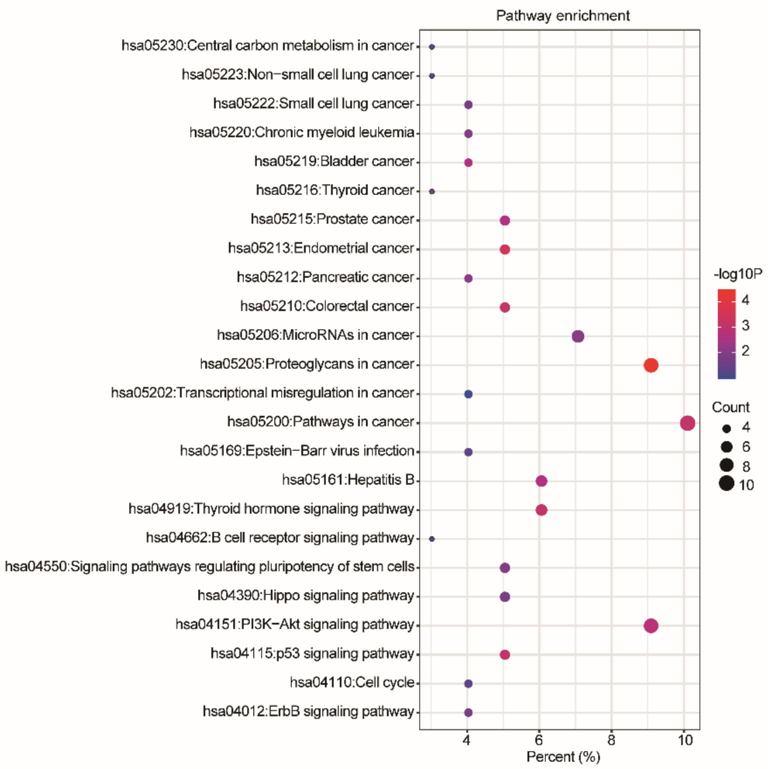

Figure S9. KEGG pathway enrichment analysis.

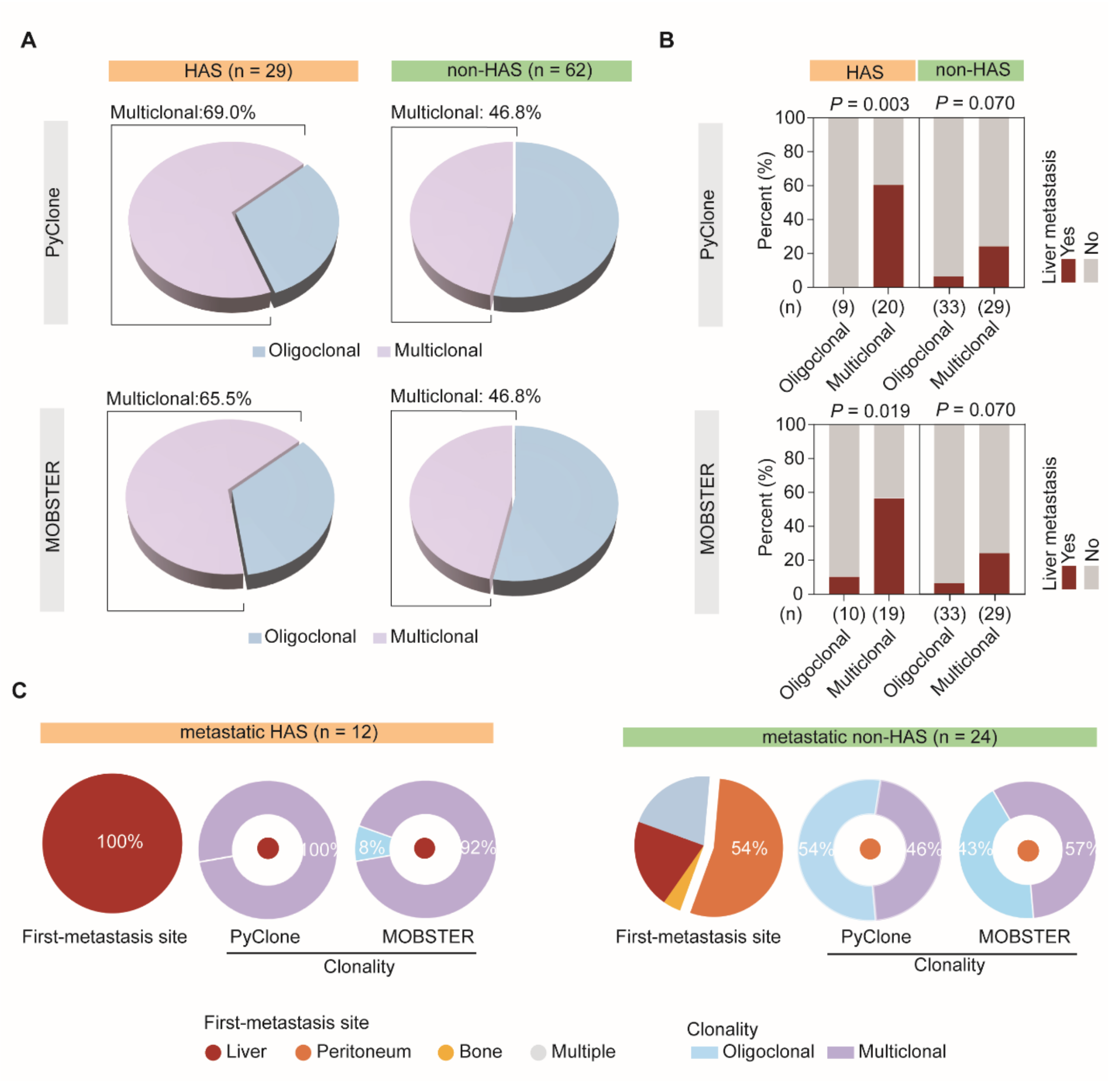

Figure S10. The clonal architecture inferred in HAS and non-HAS using PyClone and MOBSTER. **(A)** Comparison of clonal architecture between HAS and non-HAS. **(B)** Association of clonality with liver metastasis in HAS and non-HAS patients. **(C)** Association of clonality with metastatic pattern in HAS and non-HAS patients. The pie diagrams display the distribution of first-metastasis site in metastatic HAS and metastatic non-HAS patients. The circular diagrams display the distribution of clonal architecture in HAS and non-HAS patients with the most frequent metastasis site.

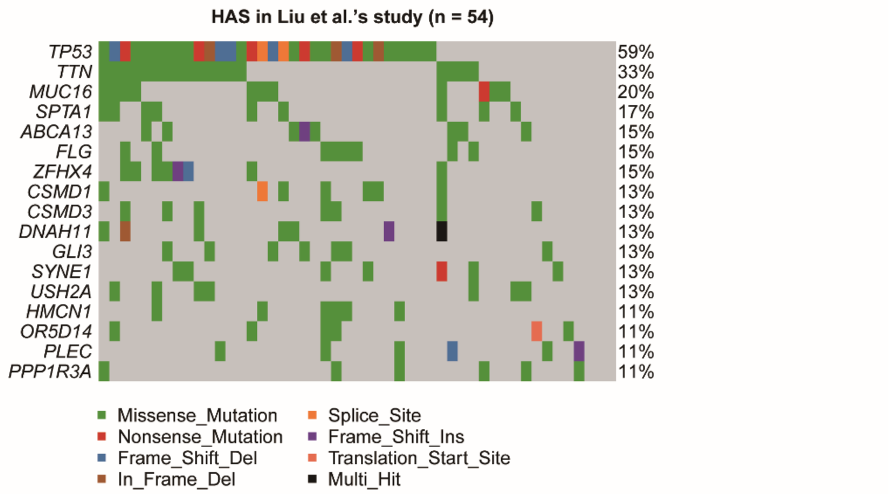

Figure S11. Frequently mutated genes of HAS in Liu et al.'s study.

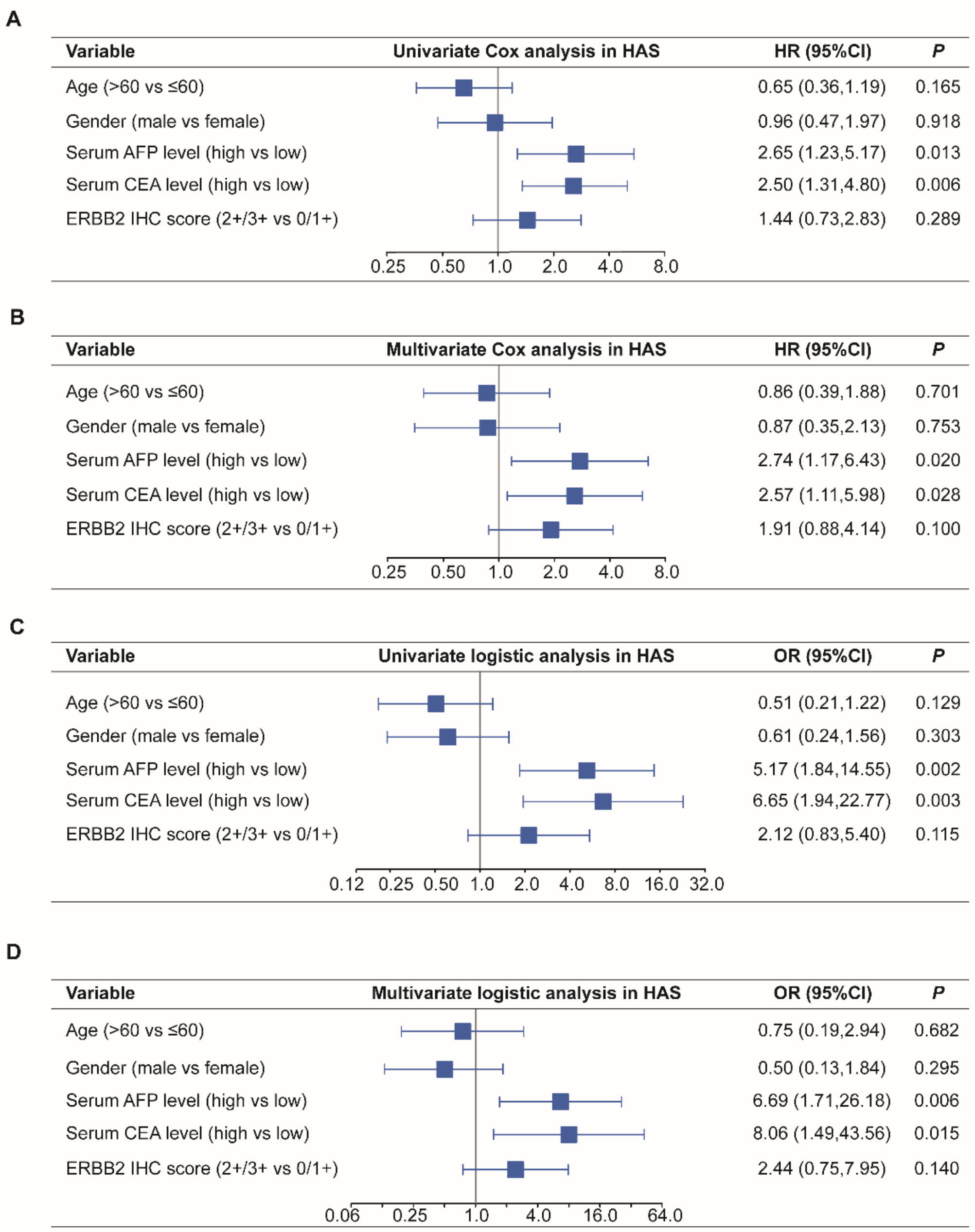

Figure S12. Forest plot showing COX analysis (A-B) for OS and logistic analysis (C-D) for liver metastasis in HAS. (A) Univariate COX analysis; **(B)** Multivariate COX analysis; **(C)** Univariate logistic analysis; **(D)** Multivariate logistic analysis. Variables include age, gender, serum AFP and CEA level, and ERBB2 IHC score. The hazard ratios or odds ratios of each variable are presented and the horizontal lines indicate the 95% confidence intervals.

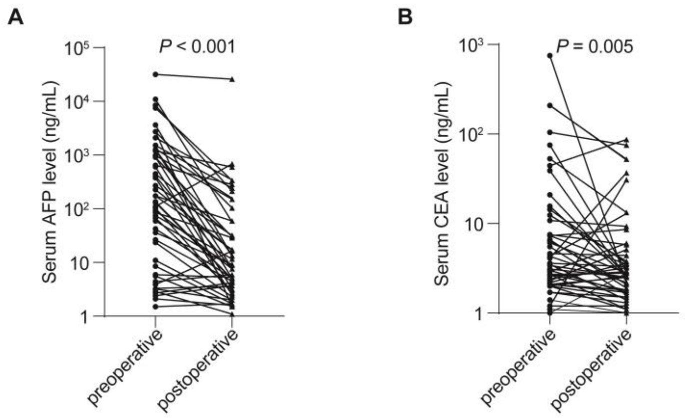

Figure S13. Comparison between preoperative and postoperative level of serum tumor markers for HAS patients receiving radical surgery. (A) AFP; **(B)** CEA.

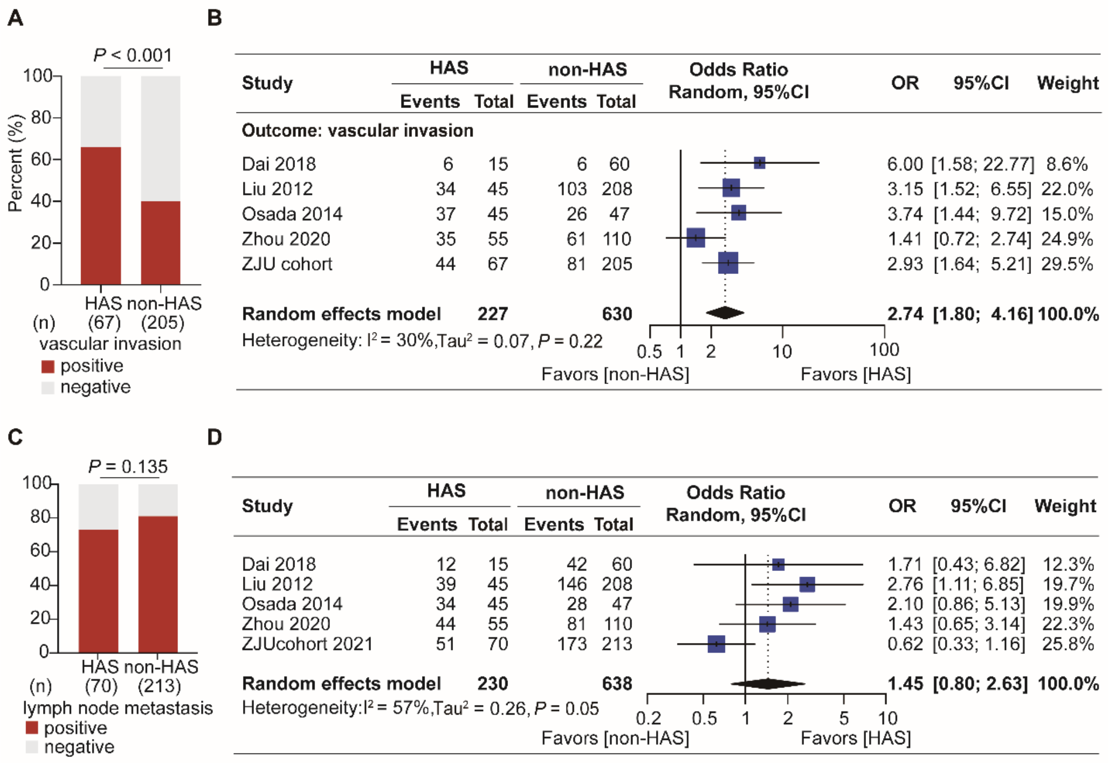

Figure S14. Comparison of clinicopathological features between HAS and non-HAS in ZJU cohort and Meta-analysis. (A-B) Comparison of vascular invasion in ZJU-cohort **(A)** and meta-analysis **(B); (C-D)** Comparison of lymph node metastasis in ZJU-cohort **(C)** and meta-analysis **(D)**.

Table S1. Comparison of clinicopathological characteristics between HAS and CGC patients

Characteristic	HAS (N = 90)	CGC (N = 973)	*P*
**Age -years**			0.755
≤60	33(36.7)	373(38.3)	
>60	57(63.3)	600(61.7)	
**Gender**			0.458
Female	24(26.7)	296(30.4)	
Male	66(73.3)	677(69.6)	
**T stage**			0.184
T1/2	22(24.4)	349(35.9)	
T3/4	48(53.3)	535(55.0)	
Unknown	20(22.2)	89(9.1)	
**N stage**			0.083
N0	19(21.1)	359(36.9)	
N1	12(13.3)	152(15.6)	
N2	19(21.2)	154(15.8)	
N3	20(22.2)	221(22.7)	
Unknown	20(22.2)	87(8.9)	
**M stage**			< 0.001
M0	59(65.6)	817(84.0)	
M1	31(34.4)	145(14.9)	
Unknown	0(0)	11(1.1)	
**AJCC stage**			< 0.001
I	9(10.0)	276(28.4)	
II	16(17.8)	219(22.5)	
III	34(37.8)	323(33.2)	
IV	31(34.4)	144(14.8)	
Unknown	0(0)	11(1.1)	

The cases with unknown data were not included in the statistical analysis. HAS, hepatoid adenocarcinoma of the stomach; CGC, conventional gastric cancer.

Table S2. Baseline characteristics of included studies in the meta-analysis

Author	Year	Country	Group	N	Age		Gender		T stage		
					<60 y	≥60 y	Male	Female	T1/2	T3/4	NA
Dai et al.	2018	China	HAS	15	8(53.3)	7(46.7)	12(80.0)	3(20.0)	5(33.3)	10(66.7)	0(0)
			non-HAS	60	32(53.3)	28(46.7)	42(70.0)	18(30.0)	14(23.3)	46(76.7)	0(0)
Liu et al.	2012	China	HAS	45	na	na	35(77.8)	10(22.2)	na	na	0(0)
			non-HAS	208	na	na	150(72.1)	58(27.9)	na	na	0(0)
Nagai et al.	1993	Japan	HAS	28	na	na	18(64.3)	10(35.7)	na	na	0(0)
			non-HAS	839	na	na	na	na	na	na	na
Osada et al.	2014	Japan	HAS	45	na	na	32(71.1)	13(28.9)	15(33.3)	30(66.7)	0(0)
			non-HAS	47	na	na	14(29.8)	33(70.2)	19(40.4)	28(59.6)	0(0)
Zhou et al.	2020	China	HAS	55	27(49.1)	28(50.9)	44(80.0)	11(20.0)	17(30.9)	38(69.1)	0(0)
			non-HAS	110	62(56.4)	48(43.6)	87(79.1)	23(20.9)	34(30.9)	76(69.1)	0(0)
ZJU cohort[*]	/	China	HAS	90	28(31.1)	62(68.9)	66(73.3)	24(26.7)	22(24.4)	48(53.3)	20(22.2)
			non-HAS	270	112(41.5)	158(58.5)	89(33.0)	181(67.0)	47(17.4)	166(61.5)	57(21.1)

Table S2. Baseline characteristics of included studies in the meta-analysis (*continued*)

Author	Group	N	Liver metastasis - n(%)			Vascular invasion - n(%)			Lymph node metastasis - n(%)			Data extraction[#]	Stage-matched[^]
			**Pos.**	**Neg.**	**NA**	**Pos.**	**Neg.**	**NA**	**Pos.**	**Neg.**	**NA**		
Dai et al.	HAS	15	8(53.3)	7(46.7)	0(0)	6(40.0)	9(60.0)	0(0)	12(80.0)	3(20.0)	0(0)	indirect	Yes
	non-HAS	60	7(11.7)	53(88.3)	0(0)	6(10.0)	54(90.0)	0(0)	42(70.0)	18(30.0)	0(0)		
Liu et al.	HAS	45	34(75.6)	11(24.4)	0(0)	34(75.6)	11(24.4)	0(0)	39(86.7)	6(13.3)	0(0)	indirect	Yes
	non-HAS	208	24(11.5)	184(88.5)	0(0)	103(49.5)	105(50.5)	0(0)	146(70.2)	62(29.8)	0(0)		
Nagai et al.	HAS	28	8(28.6)	13(46.4)	7(25.0)	24(85.7)	4(14.3)	0(0)	20(71.4)	4(14.3)	4(14.3)	indirect	No
	non-HAS	839	na	na	na	na	na	na	na	na	na		
Osada et al.	HAS	45	16(35.6)	29(64.4)	0(0)	37(82.2)	8(17.8)	0(0)	34(75.6)	11(24.4)	0(0)	indirect	No
	non-HAS	47	7(14.9)	33(70.2)	7(14.9)	26(55.3)	21(44.7)	0(0)	28(59.6)	17(36.2)	2(4.3)		
Zhou et al.	HAS	55	na	na	na	35(63.6)	20(36.4)	0(0)	44(80.0)	11(20.0)	0(0)	direct	Yes
	non-HAS	110	na	na	na	61(55.5)	49(44.5)	0(0)	81(73.6)	29(26.4)	0(0)		
ZJU cohort[*]	HAS	90	37(41.1)	53(58.9)	0(0)	44(48.9)	23(25.6)	23(25.6)	51(56.7)	19(21.1)	20(22.2)	direct	Yes
	non-HAS	270	48(17.8)	222(82.2)	0(0)	81(30.0)	124(45.9)	65(24.1)	173(64.1)	40(14.8)	57(21.1)		

[*]a clinical cohort collected from our institution; [#]The direct method of data extraction means that the survival data including the value of HRs and 95%CIs are available from the studies, whereas the indirect method means that the survival data were obtained from the email of corresponding authors or Kaplan–Meier curves in the articles. [^] "Yes" represents that non-HAS patients were staged-matched with HAS patients. Abbreviation: HAS, hepatoid adenocarcinoma of the stomach; non-HAS, non-hepatoid adenocarcinoma of the stomach; N, the number of patient; Pos., positive; Neg., negative; y, year; NA/na, not available.

Table S3. Quality assessment of included studies using Newcastle-Ottawa scale^

Study	Selection				Comparability	Outcome			Score
	Representativeness of exposed cohort	Selection of non-exposed	Ascertainment of exposure	Outcome not present at start		Assessment of outcome	Adequate follow-up	Adequacy of follow-up	
**Dai et al. 2018**	*	*	*	*	*	*	*	/	7
**Liu et al. 2012**	*	*	*	*	*	*	*	/	7
**Nagai et al. 1993**	*	*	*	*	/	*	*	/	6
**Osada et al. 2014**	*	*	*	*	/	*	*	/	6
**Zhou et al. 2020**	*	*	*	*	*	*	*	/	7
**ZJU cohort**	*	*	*	*	*	*	*	/	7

^Newcastle-Ottawa scale: study can be labeled by one star (*) for meeting each criterion, except that comparability can be labeled by a maximum of two stars (**)

Table S4. Subgroup analysis for the comparison of OS between HAS and non-HAS patients

	Study number	Pooled HR(95%CI)	***P***	Heterogeneity	
				**I^2**	***P***
**Country**					
China	4	3.16 (1.48, 6.75)	0.003	83%	< 0.01
Japan	2	2.79 (1.27, 6.12)	0.011	65%	0.09
**Data extraction**					
Direct	2	1.81 (1.23, 2.65)	0.003	0%	0.50
Indirect	4	3.79 (2.36, 6.10)	< 0.001	60%	0.06
**Stage-matched manner**					
Yes	4	3.16 (1.48, 6.75)	0.003	83%	0.01
No	2	2.79 (1.27, 6.12)	0.011	65%	0.09
**Diagnosis criteria**					
Morphology	3	1.89 (1.34, 2.67)	< 0.001	0%	0.38
Morphology and AFP production	3	4.46 (2.91, 6.83)	< 0.001	45%	0.16

Table S5. Baseline characteristics of HAS patients in the training cohort and validation cohort

	Training cohort (n = 77)	Validation cohort (n =25)	*P*
**Age-y**			0.592
≤60	26(33.8%)	7(28.0%)	
>60	51(66.2%)	18(72.0%)	
**Gender**			0.740
Female	19(24.7%)	7(28.0%)	
Male	58(75.3%)	18(72.0%)	
**Size-cm**			0.761
≤ 5.0	40(66.7%)	12(70.6%)	
> 5.0	20(33.3%)	5(29.4%)	
NA	17	8	
**Tumor location**			0.430
Antrum	38(49.4%)	12(48.0%)	
Body	19(24.7%)	9(36.0%)	
Cardia	20(26.0%)	4(16.0%)	
**Vascular invasion**			0.854
Negative	22(37.3%)	7(35.0%)	
Positive	37(62.7%)	13(65.0%)	
NA	18	5	
**Lymph node metastasis**			0.572
Negative	15(25.0%)	6(31.6%)	
Positive	45(75.0%)	13(68.4%)	
NA	17	6	
**Differentiation**			0.605
Moderate/moderate-poor	24(38.1%)	6(31.6%)	
Poor	39(61.9%)	13(68.4%)	
NA	14	6	
**AFP-ng/mL**			0.104
<173.2	38(49.4%)	17(68.0%)	
≥173.2	39(50.6%)	8(32.0%)	
**CEA-ng/mL**			0.104
<4.1	38(49.4%)	17(68.0%)	
≥4.1	39(50.6%)	8(32.0%)	
**CA19-9-U/mL**			0.104
<9.6	38(49.4%)	17(68.0%)	
≥9.6	39(50.6%)	8(32.0%)	
**CA125-U/mL**			0.202
<13.4	38(49.4%)	16(64.0%)	
≥13.4	39(50.6%)	9(36.0%)	
**SF-ng/mL**			0.202
<99.5	38(49.4%)	16(64.0%)	

≥99.5	39(50.6%)	9(36.0%)	
**WBC-10^9/L**			0.104
<6.3	38(49.4%)	17(68.0%)	
≥6.3	39(50.6%)	8(32.0%)	
**NEUT-%**			0.130
<64.3	38(49.4%)	8(32.0%)	
≥64.3	39(50.6%)	17(68.0%)	
**LYM-%**			0.064
<25.5	38(49.4%)	18(72.0%)	
≥25.5	39(50.6%)	7(28.0%)	
**NLR**			0.062
<2.49	38(49.4%)	7(28.0%)	
≥2.49	39(50.6%)	18(72.0%)	
**MO-%**			0.818
<7.5	38(49.4%)	13(52.0%)	
≥7.5	39(50.6%)	12(48.0%)	
**ALB-g/L**			0.732
<39.3	37(48.1%)	13(52.0%)	
≥39.3	40(51.9%)	12(48.0%)	
**GLB-g/L**			0.642
<25.8	38(49.4%)	11(44.0%)	
≥25.8	39(50.6%)	14(56.0%)	
**AGR**			0.202
<1.55	38(49.4%)	16(64.0%)	
≥1.55	39(50.6%)	9(36.0%)	
**ALT-U/L**			0.063
<19	37(48.1%)	18(72.0%)	
≥19	40(51.9%)	7(28.0%)	
**AST-U/L**			0.299
<24	37(48.1%)	15(60.0%)	
≥24	40(51.9%)	10(40.0%)	
**ALP-U/L**			0.416
<80	38(49.4%)	10(40.0%)	
≥80	39(50.6%)	15(60.0%)	
**TBil-μmol/L**			0.483
<10	37(48.1%)	10(40.0%)	
≥10	40(51.9%)	15(60.0%)	
**DBil-μmol/L**			0.062
<3.7	38(49.4%)	7(28.0%)	
≥3.7	39(50.6%)	18(72.0%)	
**ERBB2 IHC**			0.573
-/+	38(55.9%)	15(62.5%)	
++/+++	30(44.1%)	9(37.5%)	
NA	9	1	

**Liver metastasis**			0.425
Yes	47(61.0%)	13(52.0%)	
No	30(39.0%)	12(48.0%)	

Table S6. Univariate analysis for factors associated with liver metastasis of HAS patients in the training cohort

	No liver metastasis (n = 47)	Liver metastasis (n = 30)	*P*
**Age-y**			0.056
≤60	12(25.5)	14(46.7)	
>60	35(74.5)	16(53.3)	
**Gender**			0.746
Female	11(23.4)	8(26.7)	
Male	36(76.6)	22(73.3)	
**Size-cm**			0.829
≤ 5.0	31(67.4)	9(64.3)	
> 5.0	15(32.6)	5(35.7)	
NA	1	16	
**Tumor location**			0.537
Antrum	23(48.9)	15(50.0)	
Body	10(21.3)	9(30.0)	
Cardia	14(29.8)	6(20.0)	
**Vascular invasion**			0.260
Negative	15(33.3)	7(50.0)	
Positive	30(66.7)	7(50.0)	
NA	2	16	
**Lymph node metastasis**			0.155
Negative	14(30.4)	1(7.1)	
Positive	32(69.6)	13(92.9)	
NA	1	16	
**Differentiation**			0.759
Moderate/moderate-poor	17(37.0)	7(41.2)	
Poor	29(63.0)	10(58.8)	
NA	1	13	
**AFP-ng/mL**			0.075
<173.2	27(57.4)	11(36.7)	
≥173.2	20(42.6)	19(63.3)	
**CEA-ng/mL**			0.707
<4.1	24(51.1)	14(46.7)	
≥4.1	23(48.9)	16(53.3)	
**CA19-9-U/mL**			0.007
<9.6	29(61.7)	9(30.0)	
≥9.6	18(38.3)	21(70.0)	
**CA125-U/mL**			0.025
<13.4	28(59.6)	10(33.3)	
≥13.4	19(40.4)	20(66.7)	
**SF-ng/mL**			0.707
<99.5	24(51.1)	14(46.7)	

≥99.5	23(48.9)	16(53.3)	
**WBC-10^9/L**			0.577
<6.3	22(46.8)	16(53.3)	
≥6.3	25(53.2)	14(46.7)	
**NEUT-%**			0.577
<64.3	22(46.8)	16(53.3)	
≥64.3	25(53.2)	14(46.7)	
**LYM-%**			0.927
<25.5	23(48.9)	15(50.0)	
≥25.5	24(51.1)	15(50.0)	
**NLR**			0.707
<2.49	24(51.1)	14(46.7)	
≥2.49	23(48.9)	16(53.3)	
**MO-%**			0.399
<7.5	25(53.2)	13(43.3)	
≥7.5	22(46.8)	17(56.7)	
**ALB-g/L**			0.459
<39.3	21(44.7)	16(53.3)	
≥39.3	26(55.3)	14(46.7)	
**GLB-g/L**			0.025
<25.8	28(59.6)	10(33.3)	
≥25.8	19(40.4)	20(66.7)	
**AGR**			0.135
<1.55	20(42.6)	18(60.0)	
≥1.55	27(57.4)	12(40.0)	
**ALT-U/L**			0.508
<19	24(51.1)	13(43.3)	
≥19	23(48.9)	17(56.7)	
**AST-U/L**			0.110
<24	26(55.3)	11(36.7)	
≥24	21(44.7)	19(63.3)	
**ALP-U/L**			0.001
<80	30(63.8)	8(26.7)	
≥80	17(36.2)	22(73.3)	
**TBil-μmol/L**			0.508
<10	24(51.1)	13(43.3)	
≥10	23(48.9)	17(56.7)	
**DBil-μmol/L**			0.707
<3.7	24(51.1)	14(46.7)	
≥3.7	23(48.9)	16(53.3)	
**ERBB2 IHC**			0.141
-/+	28(62.2)	10(43.5)	
++/+++	17(37.8)	13(56.5)	
NA	2	7	

Table S7. Multivariate Logistic regression analysis for factors associated with liver metastasis of HAS patients in the training cohort

	B	OR(95%CI)	*P*
**Age-y**			
≤60	reference		
>60	-0.718	0.49(0.15-1.59)	0.234
**AFP-ng/mL**			
<173.2	reference		
≥173.2	1.108	3.03(0.91-10.06)	0.070
**CA19-9-U/mL**			
<9.6	reference		
≥9.6	1.385	4.00(1.25-12.82)	0.020
**CA125-U/mL**			
<13.4	reference		
≥13.4	0.755	2.13(0.69-6.56)	0.189
**GLB-g/L**			
<25.8	reference		
≥25.8	0.184	1.20(0.37-3.92)	0.761
**ALP-U/L**			
<80	reference		
≥80	1.266	3.55(1.05-12.03)	0.042

Table S8. Sequencing depth of WES performing on the genomic DNA of tumor and normal sample in HAS and non-HAS

Cohort	ID-normal	sequencing depth	ID-tumor	sequencing depth
HAS	HAS13_N	92.43	HAS13_T	169.63
HAS	HAS6_N	100.08	HAS6_T	240.42
HAS	HAS11_N	144.8	HAS11_T	113.05
HAS	HAS14_N	80.89	HAS14_T	211.74
HAS	HAS12_N	111.73	HAS12_T	189.39
HAS	HAS17_N	182.05	HAS17_T	157.2
HAS	HAS8_N	108.95	HAS8_T	197.01
HAS	HAS15_N	81.76	HAS15_T	222.96
HAS	HAS5_N	91.75	HAS5_T	158.77
HAS	HAS9_N	86.91	HAS9_T	152.04
HAS	HAS24_N	85.81	HAS24_T	109.71
HAS	HAS16_N	100.16	HAS16_T	75.51
HAS	HAS25_N	109.54	HAS25_T	222.28
HAS	HAS7_N	198.83	HAS7_T	177.49
HAS	HAS4_N	84.37	HAS4_T	102.98
HAS	HAS2_N	86.83	HAS2_T	225.82
HAS	HAS20_N	69.23	HAS20_T	74.41
HAS	HAS26_N	72.87	HAS26_T	157.08
HAS	HAS18_N	102.15	HAS18_T	189.18
HAS	HAS19_N	145.71	HAS19_T	86.71
HAS	HAS1_N	95.53	HAS1_T	86.37
HAS	HAS3_N	104.32	HAS3_T	237.01
HAS	HAS23_N	114.55	HAS23_T	95.1
HAS	HAS22_N	98.24	HAS22_T	100.49
HAS	HAS10_N	78.61	HAS10_T	92.65
HAS	HAS21_N	100.76	HAS21_T	138.25
HAS	HAS27_N	203	HAS27_T	453
HAS	HAS30_N	266	HAS30_T	365
HAS	HAS29_N	192	HAS29_T	190
HAS	HAS28_N	200	HAS28_T	177
non-HAS	non-HAS1_N	93	non-HAS1_T	360
non-HAS	non-HAS2_N	109	non-HAS2_T	301
non-HAS	non-HAS3_N	225	non-HAS3_T	402
non-HAS	non-HAS4_N	225	non-HAS4_T	620
non-HAS	non-HAS5_N	238	non-HAS5_T	485
non-HAS	non-HAS6_N	179	non-HAS6_T	473
non-HAS	non-HAS7_N	136	non-HAS7_T	472
non-HAS	non-HAS8_N	290	non-HAS8_T	637
non-HAS	non-HAS9_N	207	non-HAS9_T	192
non-HAS	non-HAS10_N	249	non-HAS10_T	302
non-HAS	non-HAS11_N	199	non-HAS11_T	180

non-HAS	non-HAS12_N	303	non-HAS12_T	207
non-HAS	non-HAS13_N	281	non-HAS13_T	301
non-HAS	non-HAS14_N	225	non-HAS14_T	265
non-HAS	non-HAS15_N	120	non-HAS15_T	382
non-HAS	non-HAS16_N	146	non-HAS16_T	340
non-HAS	non-HAS17_N	102	non-HAS17_T	303
non-HAS	non-HAS18_N	120	non-HAS18_T	327
non-HAS	non-HAS19_N	140	non-HAS19_T	414
non-HAS	non-HAS20_N	142	non-HAS20_T	347
non-HAS	non-HAS21_N	126	non-HAS21_T	428
non-HAS	non-HAS22_N	149	non-HAS22_T	496
non-HAS	non-HAS23_N	149	non-HAS23_T	565
non-HAS	non-HAS24_N	183	non-HAS24_T	169
non-HAS	non-HAS25_N	165	non-HAS25_T	162
non-HAS	non-HAS26_N	161	non-HAS26_T	159
non-HAS	non-HAS27_N	168	non-HAS27_T	188
non-HAS	non-HAS28_N	185	non-HAS28_T	166
non-HAS	non-HAS29_N	179	non-HAS29_T	163
non-HAS	non-HAS30_N	167	non-HAS30_T	174
non-HAS	non-HAS31_N	174	non-HAS31_T	164
non-HAS	non-HAS32_N	159	non-HAS32_T	160
non-HAS	non-HAS33_N	151	non-HAS33_T	154
non-HAS	non-HAS34_N	155	non-HAS34_T	158
non-HAS	non-HAS35_N	170	non-HAS35_T	169
non-HAS	non-HAS36_N	165	non-HAS36_T	176
non-HAS	non-HAS37_N	164	non-HAS37_T	163
non-HAS	non-HAS38_N	160	non-HAS38_T	174
non-HAS	non-HAS39_N	194	non-HAS39_T	185
non-HAS	non-HAS40_N	172	non-HAS40_T	146
non-HAS	non-HAS41_N	171	non-HAS41_T	160
non-HAS	non-HAS42_N	157	non-HAS42_T	157
non-HAS	non-HAS43_N	310	non-HAS43_T	246
non-HAS	non-HAS44_N	321	non-HAS44_T	306
non-HAS	non-HAS45_N	238	non-HAS45_T	262
non-HAS	non-HAS46_N	373	non-HAS46_T	268
non-HAS	non-HAS47_N	257	non-HAS47_T	143
non-HAS	non-HAS48_N	158	non-HAS48_T	119
non-HAS	non-HAS49_N	127	non-HAS49_T	545
non-HAS	non-HAS50_N	126	non-HAS50_T	640
non-HAS	non-HAS51_N	141	non-HAS51_T	585
non-HAS	non-HAS52_N	155	non-HAS52_T	516
non-HAS	non-HAS53_N	154	non-HAS53_T	606
non-HAS	non-HAS54_N	174	non-HAS54_T	557
non-HAS	non-HAS55_N	103	non-HAS55_T	531

non-HAS	non-HAS56_N	107	non-HAS56_T	382
non-HAS	non-HAS57_N	110	non-HAS57_T	443
non-HAS	non-HAS58_N	122	non-HAS58_T	386
non-HAS	non-HAS59_N	165	non-HAS59_T	469
non-HAS	non-HAS60_N	120	non-HAS60_T	338
non-HAS	non-HAS61_N	121	non-HAS61_T	297
non-HAS	non-HAS62_N	150	non-HAS62_T	264
non-HAS	non-HAS63_N	135	non-HAS63_T	371

Table S9. Comparison of frequently mutated genes between HAS and non-HAS

Gene	HAS (n = 30)		Non-HAS (n = 63)		$P^{\#}$
	Mut	Wt	Mut	Wt	
TP53	20	10	32	31	0.150
TTN	10	20	26	37	0.463
PCLO	9	21	6	57	0.017
CSMD3	9	21	13	50	0.320
OBSCN	8	22	6	57	0.059
COL11A1	7	23	2	61	0.005
ABCA13	6	24	1	62	0.004
MUC6	6	24	3	60	0.029
KMT2C	6	24	6	57	0.192
MUC4	6	24	7	56	0.338
FSIP2	6	24	10	53	0.622
MUC16	6	24	12	51	0.913
MDC1	5	25	0	63	0.003
BRCA2	5	25	2	61	0.034
MYO15A	5	25	3	60	0.106
COL5A3	5	25	5	58	0.283
FLG	5	25	5	58	0.283
MYT1L	5	25	5	58	0.283
ZFHX4	5	25	6	57	0.324
LRP1B	5	25	16	47	0.347
GPR112	4	26	0	63	0.009
MYCBP2	4	26	0	63	0.009
PRDM1	4	26	0	63	0.009
TCHH	4	26	0	63	0.009
ABCB4	4	26	1	62	0.036
BCOR	4	26	1	62	0.036
HUWE1	4	26	1	62	0.036
MGA	4	26	1	62	0.036
PCDHA11	4	26	1	62	0.036
SSC5D	4	26	1	62	0.036
TPTE	4	26	1	62	0.036
SYNE1	4	26	20	43	0.058
LAMA1	4	26	2	61	0.083
LRRC7	4	26	2	61	0.083
ZNF804A	4	26	2	61	0.083
ASPM	4	26	3	60	0.207
FLG2	4	26	3	60	0.207
PCDH15	4	26	3	60	0.207
DNAH8	4	26	4	59	0.267
PKHD1	4	26	4	59	0.267
XIRP2	4	26	4	59	0.267

LRP2	4	26	5	58	0.463
CCDC168	4	26	12	51	0.495
FAT3	4	26	6	57	0.722
FCGBP	4	26	6	57	0.722
HMCN1	3	27	11	52	0.537
PREX2	3	27	9	54	0.745
FAT4	3	27	8	55	1.000
NEB	3	27	8	55	1.000
RYR1	2	28	8	55	0.492
NRXN2	2	28	7	56	0.713
PXDN	2	28	7	56	0.713
SPTA1	1	29	11	52	0.095
LAMA5	1	29	9	54	0.159
ARID1A	1	29	7	56	0.430

[#]statistical significance was determined by chi-q test or Fisher's test. Mut, mutation; Wt, wild type.

Table S10. Comparison of frequently mutated genes between HAS and TCGA-LIHC

Gene	HAS (n = 30)		TCGA-LIHC (n = 355)		$P^{\#}$
	Mut	Wt	Mut	Wt	
TP53	20	10	112	243	< 0.001
TTN	10	20	99	256	0.525
CSMD3	9	21	31	324	< 0.001
PCLO	9	21	34	321	0.003
OBSCN	8	22	30	325	0.005
COL11A1	7	23	21	334	0.003
FSIP2	6	24	10	345	0.001
MUC6	6	24	12	343	0.001
KMT2C	6	24	20	335	0.010
MUC4	6	24	25	330	0.025
ABCA13	6	24	30	325	0.049
MUC16	6	24	52	303	0.427
MDC1	5	25	6	349	0.001
COL5A3	5	25	9	346	0.002
MYO15A	5	25	10	345	0.004
BRCA2	5	25	11	344	0.005
MYT1L	5	25	13	342	0.009
ZFHX4	5	25	20	335	0.036
FLG	5	25	26	329	0.081
LRP1B	5	25	32	323	0.190
BCOR	4	26	2	353	< 0.001
PCDHA11	4	26	2	353	< 0.001
PRDM1	4	26	4	351	0.002
ABCB4	4	26	5	350	0.003
CCDC168	4	26	6	349	0.005
FLG2	4	26	7	348	0.007
SSC5D	4	26	7	348	0.007
LRRC7	4	26	9	346	0.013
TPTE	4	26	9	346	0.013
ASPM	4	26	10	345	0.017
ZNF804A	4	26	11	344	0.022
FCGBP	4	26	12	343	0.028
GPR112	4	26	12	343	0.028
HUWE1	4	26	12	343	0.028
MGA	4	26	12	343	0.028
PCDH15	4	26	15	340	0.051
LAMA1	4	26	16	339	0.060
MYCBP2	4	26	16	339	0.060
TCHH	4	26	16	339	0.060
PKHD1	4	26	17	338	0.070
LRP2	4	26	18	337	0.081

DNAH8	4	26	19	336	0.093
SYNE1	4	26	22	333	0.133
FAT3	4	26	25	330	0.266
XIRP2	4	26	28	327	0.297
RYR2	2	28	37	318	0.754
APOB	1	29	38	317	0.341
CTNNB1	0	30	97	258	< 0.001
ALB	0	30	42	313	0.060

#statistical significance was determined by chi-q test or Fisher's test. Mut, mutation; Wt, wild type.

Table S11. Comparison of four TCGA subtypes between HAS and non-HAS

TCGA subtypes	HAS	Non-HAS	*P*
CIN	17(56.7)	24(38.1)	
GS	13(43.3)	34(54.0)	0.219
MSI	0(0.0)	2(3.2)	
EBV	0(0.0)	3(4.8)	

CIN, chromosomal instability; GS, genomic stability; MSI, microsatellite instability; EBV, Epstein-Barr virus.

Table S12. Comparison of frequent and significant CNVs among HAS, non-HAS, and TCGA-LIHC

CNV	HAS		non-HAS		*P*	TCGA-LIHC		*P*
	Altered	No altered	Altered	No altered		Altered	No altered	
AMP in 17q12	9(30.0)	21(70.0)	8(12.7)	55(87.3)	0.044	7(2.0)	348(98.0)	<0.001
AMP in 19q12	8(26.7)	22(73.3)	5(7.9)	58(92.1)	0.024	8(2.3)	347(97.7)	<0.001
AMP in 20q13.12	4(13.3)	26(86.7)	5(7.9)	58(92.1)	0.463	6(1.7)	349(98.3)	0.005
AMP in 3q29	7(23.3)	23(76.7)	3(4.8)	60(95.2)	0.012	8(2.3)	347(97.7)	<0.001
AMP in 6p21.33	5(16.7)	25(83.3)	3(4.8)	60(95.2)	0.106	15(4.2)	340(95.8)	0.003
AMP in 8q21.2	5(16.7)	25(83.3)	23(36.5)	40(63.5)	0.106	49(13.8)	306(86.2)	0.665
AMP in 8q24.21	7(23.3)	23(76.7)	3(4.8)	60(95.2)	0.012	63(17.7)	292(82.3)	0.446
DEL in 16p13.3	4(13.3)	26(86.7)	0(0.0)	63(100.0)	0.009	3(0.8)	352(99.2)	0.001
DEL in 4q35.2	5(16.7)	25(83.3)	5(7.9)	58(92.1)	>0.999	14(3.9)	341(96.1)	0.002

Statistics analysis was conducted using chi-q test or Fisher's exact test to compare the frequency of CNVs between HAS and non-HAS or TCGA-LIHC. AMP, amplification; DEL, deletion.

Table S13. The tumor clonality of each sample in HAS and non-HAS

HAS			Non-HAS					
ID	Clonal number	Type	ID	Clonal number	Type	ID	Clonal number	Type
HAS1	1	Mono	non-HAS10	1	Mono	non-HAS28	2	Minor
HAS11	1	Mono	non-HAS11	1	Mono	non-HAS33	2	Minor
HAS4	1	Mono	non-HAS15	1	Mono	non-HAS54	2	Minor
HAS22	1	Mono	non-HAS17	1	Mono	non-HAS56	2	Minor
HAS5	1	Mono	non-HAS18	1	Mono	non-HAS49	2	Minor
HAS6	1	Mono	non-HAS19	1	Mono	non-HAS44	2	Minor
HAS7	2	Minor	non-HAS20	1	Mono	non-HAS8	2	Minor
HAS10	2	Minor	non-HAS24	1	Mono	non-HAS13	3	Complex
HAS18	2	Minor	non-HAS25	1	Mono	non-HAS26	3	Complex
HAS16	2	Bio	non-HAS27	1	Mono	non-HAS39	3	Complex
HAS23	2	Bio	non-HAS29	1	Mono	non-HAS57	3	Complex
HAS2	2	Bio	non-HAS30	1	Mono	non-HAS60	3	Complex
HAS25	3	Complex	non-HAS31	1	Mono	non-HAS6	3	Complex
HAS29	3	Complex	non-HAS32	1	Mono	non-HAS22	3	Complex
HAS17	3	Complex	non-HAS34	1	Mono	non-HAS62	4	Complex
HAS9	3	Complex	non-HAS35	1	Mono	non-HAS7	4	Complex
HAS20	3	Complex	non-HAS36	1	Mono	non-HAS16	4	Complex
HAS19	3	Complex	non-HAS37	1	Mono	non-HAS53	4	Complex
HAS15	3	Complex	non-HAS38	1	Mono	non-HAS63	4	Complex
HAS3	4	Complex	non-HAS40	1	Mono	non-HAS45	4	Complex
HAS24	4	Complex	non-HAS42	1	Mono	non-HAS48	5	Complex
HAS28	4	Complex	non-HAS43	1	Mono	non-HAS61	5	Complex
HAS8	4	Complex	non-HAS46	1	Mono	non-HAS1	5	Complex
HAS12	5	Complex	non-HAS47	1	Mono	non-HAS55	5	Complex
HAS26	6	Complex	non-HAS52	1	Mono	non-HAS2	6	Complex
HAS27	6	Complex	non-HAS58	1	Mono	non-HAS14	6	Complex
HAS13	7	Complex	non-HAS9	2	Bio	non-HAS51	6	Complex
HAS30	8	Complex	non-HAS4	2	Bio	non-HAS59	6	Complex
HAS14	8	Complex	non-HAS3	2	Bio	non-HAS23	7	Complex

Table S14. Clonal architecture of each sample identified using SciClone, PyClone, and MOBSTER

Group	Sample ID	Clonal architecture		
		**SciClone**	**PyClone**	**MOBSTER**
HAS	HAS20	Multiclonal	Multiclonal	Multiclonal
HAS	HAS25	Multiclonal	Multiclonal	Multiclonal
HAS	HAS27	Multiclonal	Multiclonal	Multiclonal
HAS	HAS13	Multiclonal	Multiclonal	Oligoclonal
HAS	HAS2	Multiclonal	Multiclonal	Multiclonal
HAS	HAS16	Multiclonal	Multiclonal	Multiclonal
HAS	HAS29	Multiclonal	Multiclonal	Multiclonal
HAS	HAS24	Multiclonal	Multiclonal	Multiclonal
HAS	HAS12	Multiclonal	Multiclonal	Multiclonal
HAS	HAS30	Multiclonal	Multiclonal	Multiclonal
HAS	HAS14	Multiclonal	Multiclonal	Multiclonal
HAS	HAS4	Oligoclonal	Multiclonal	Multiclonal
HAS	HAS15	Multiclonal	Multiclonal	Multiclonal
HAS	HAS8	Multiclonal	Multiclonal	Multiclonal
HAS	HAS28	Multiclonal	Multiclonal	Multiclonal
HAS	HAS19	Multiclonal	Multiclonal	Multiclonal
HAS	HAS9	Multiclonal	Multiclonal	Multiclonal
HAS	HAS3	Multiclonal	Multiclonal	Multiclonal
HAS	HAS26	Multiclonal	Multiclonal	Multiclonal
HAS	HAS7	Oligoclonal	Oligoclonal	Multiclonal
HAS	HAS10	Oligoclonal	Multiclonal	Oligoclonal
HAS	HAS23	Multiclonal	Oligoclonal	Oligoclonal
HAS	HAS17	Multiclonal	Oligoclonal	Oligoclonal
HAS	HAS1	Oligoclonal	Oligoclonal	Oligoclonal
HAS	HAS18	Oligoclonal	Oligoclonal	Oligoclonal
HAS	HAS11	Oligoclonal	Oligoclonal	Oligoclonal
HAS	HAS22	Oligoclonal	Oligoclonal	Oligoclonal
HAS	HAS5	Oligoclonal	Oligoclonal	Oligoclonal
HAS	HAS6	Oligoclonal	Oligoclonal	Oligoclonal
non-HAS	non-HAS7	Multiclonal	Multiclonal	Multiclonal
non-HAS	non-HAS13	Multiclonal	Multiclonal	Multiclonal
non-HAS	non-HAS14	Multiclonal	Multiclonal	Multiclonal
non-HAS	non-HAS16	Multiclonal	Multiclonal	Multiclonal
non-HAS	non-HAS19	Multiclonal	Multiclonal	Multiclonal
non-HAS	non-HAS21	Multiclonal	Multiclonal	Multiclonal
non-HAS	non-HAS23	Multiclonal	Multiclonal	Multiclonal
non-HAS	non-HAS26	Multiclonal	Multiclonal	Multiclonal
non-HAS	non-HAS39	Multiclonal	Multiclonal	Multiclonal
non-HAS	non-HAS50	Multiclonal	Multiclonal	Multiclonal
non-HAS	non-HAS53	Multiclonal	Multiclonal	Multiclonal

non-HAS	non-HAS42	Oligoclonal	Multiclonal	Multiclonal
non-HAS	non-HAS49	Oligoclonal	Multiclonal	Multiclonal
non-HAS	non-HAS15	Multiclonal	Oligoclonal	Multiclonal
non-HAS	non-HAS62	Multiclonal	Multiclonal	Oligoclonal
non-HAS	non-HAS2	Multiclonal	Multiclonal	Oligoclonal
non-HAS	non-HAS17	Multiclonal	Oligoclonal	Oligoclonal
non-HAS	non-HAS4	Oligoclonal	Oligoclonal	Oligoclonal
non-HAS	non-HAS9	Oligoclonal	Oligoclonal	Oligoclonal
non-HAS	non-HAS10	Oligoclonal	Oligoclonal	Oligoclonal
non-HAS	non-HAS12	Oligoclonal	Oligoclonal	Oligoclonal
non-HAS	non-HAS18	Oligoclonal	Oligoclonal	Oligoclonal
non-HAS	non-HAS25	Oligoclonal	Oligoclonal	Oligoclonal
non-HAS	non-HAS27	Oligoclonal	Oligoclonal	Oligoclonal
non-HAS	non-HAS28	Oligoclonal	Oligoclonal	Oligoclonal
non-HAS	non-HAS29	Oligoclonal	Oligoclonal	Oligoclonal
non-HAS	non-HAS31	Oligoclonal	Oligoclonal	Oligoclonal
non-HAS	non-HAS32	Oligoclonal	Oligoclonal	Oligoclonal
non-HAS	non-HAS33	Oligoclonal	Oligoclonal	Oligoclonal
non-HAS	non-HAS34	Oligoclonal	Oligoclonal	Oligoclonal
non-HAS	non-HAS35	Oligoclonal	Oligoclonal	Oligoclonal
non-HAS	non-HAS36	Oligoclonal	Oligoclonal	Oligoclonal
non-HAS	non-HAS37	Oligoclonal	Oligoclonal	Oligoclonal
non-HAS	non-HAS40	Oligoclonal	Oligoclonal	Oligoclonal
non-HAS	non-HAS46	Oligoclonal	Oligoclonal	Oligoclonal
non-HAS	non-HAS52	Oligoclonal	Oligoclonal	Oligoclonal
non-HAS	non-HAS54	Oligoclonal	Oligoclonal	Oligoclonal
non-HAS	non-HAS56	Oligoclonal	Oligoclonal	Oligoclonal
non-HAS	non-HAS51	Multiclonal	Multiclonal	Multiclonal
non-HAS	non-HAS48	Multiclonal	Multiclonal	Multiclonal
non-HAS	non-HAS3	Oligoclonal	Oligoclonal	Oligoclonal
non-HAS	non-HAS57	Multiclonal	Multiclonal	Multiclonal
non-HAS	non-HAS59	Multiclonal	Multiclonal	Multiclonal
non-HAS	non-HAS60	Multiclonal	Multiclonal	Multiclonal
non-HAS	non-HAS47	Oligoclonal	Oligoclonal	Multiclonal
non-HAS	non-HAS11	Oligoclonal	Oligoclonal	Oligoclonal
non-HAS	non-HAS61	Multiclonal	Multiclonal	Multiclonal
non-HAS	non-HAS63	Multiclonal	Multiclonal	Multiclonal
non-HAS	non-HAS58	Oligoclonal	Multiclonal	Oligoclonal
non-HAS	non-HAS30	Oligoclonal	Oligoclonal	Multiclonal
non-HAS	non-HAS20	Oligoclonal	Oligoclonal	Oligoclonal
non-HAS	non-HAS24	Oligoclonal	Oligoclonal	Oligoclonal
non-HAS	non-HAS43	Oligoclonal	Oligoclonal	Oligoclonal
non-HAS	non-HAS44	Oligoclonal	Oligoclonal	Oligoclonal
non-HAS	non-HAS1	Multiclonal	Multiclonal	Multiclonal

non-HAS	non-HAS5	Multiclonal	Multiclonal	Multiclonal
non-HAS	non-HAS6	Multiclonal	Multiclonal	Multiclonal
non-HAS	non-HAS22	Multiclonal	Multiclonal	Multiclonal
non-HAS	non-HAS45	Multiclonal	Multiclonal	Multiclonal
non-HAS	non-HAS55	Multiclonal	Multiclonal	Multiclonal
non-HAS	non-HAS8	Oligoclonal	Oligoclonal	Oligoclonal
non-HAS	non-HAS38	Oligoclonal	Oligoclonal	Oligoclonal

Table S15. Target therapies for gene alterations and relevant clinical trials or preclinical studies in cancer therapy

Gene	Drug	Cancer type	Study ID	Phase
BCL6	FX1	Lymphoma	PMID: 27482887	Preclinical
BIRC3	LCL161	Non-small cell lung cancer	PMID: 27737687	Preclinical
BRCA2	Niraparib	Non-small cell lung cancer	NCT04475939	Phase 3
CCND1	Abemaciclib	Breast cancer	NCT04584853	Phase 3
CDK12	SR-4835,<br>THZ531	Breast cancer,<br>Ewing sarcoma	PMID: 31715127<br>PMID: 33945934	Preclinical
EED	LG1980	Prostate cancer	PMID: 34077206	Preclinical
ERBB2	Trastuzumab	Breast cancer, Gastric cancer	/	FDA approved
ERCC2	Carboplatin	Bladder Cancer	PMID: 32984035	Preclinical
IL7R	CYT-107	Bladder Cancer	NCT03513952	Phase 2
MET	Crizotinib	Non-small cell lung cancer	/	FDA approved
MYC	MYCi361	Solid tumors	PMID: 31679823	Preclinical

Table S16. Comparison of clinicopathological characteristics of HAS patients between ZJU-WES cohort and Liu et al. cohort

Characteristic	HAS		*P*
	**ZJU-WES cohort (N = 30)**	**Liu et al. cohort (N = 55)**	
**Age -years**			0.061
≤ 60	10(33.3)	30(54.5)	
> 60	20(66.7)	25(45.5)	
**Gender**			0.829
Female	7(23.3)	14(25.5)	
Male	23(76.7)	41(74.5)	
**T stage**			0.418
T1	2(7.1)	7(12.7)	
T2	7(25.0)	10(18.2)	
T3	10(35.7)	27(49.1)	
T4	9(32.1)	11(20.0)	
Unknown [a]	2	0	
**N stage**			0.260
N0	7(25.0)	10(18.2)	
N1	3(10.7)	16(29.1)	
N2	11(39.3)	15(27.3)	
N3	7(25.0)	14(25.5)	
Unknown [a]	2	0	
**M stage**			< 0.001[b]
M0	21(70.0)	54(98.2)	
M1	9(30.0)	1(1.8)	
**AJCC stage**			0.001
I	4(13.3)	8(14.5)	
II	4(13.3)	18(32.7)	
III	13(43.3)	28(50.9)	
IV	9(30.0)	1(1.8)	
**Location**			0.255
Antrum	13(43.3)	26(47.3)	
Body	8(26.7)	7(12.7)	
GEJ/Cardia	9(30.0)	22(40.0)	
**Serum AFP level -ng/mL**			0.005[c]
Median	195.9	20.9	
Range	2.7 – 41253.3	1.4 – 7335.0	
**Group by AFP -ng/mL**			0.001[b]
≤ 20.0	4(13.3)	21(50.0)	
> 20.0	26(86.7)	21(50.0)	
Unknown [a]	0	13	

[a] The cases with unknown data were not included in the statistical analysis. [b] Statistical analysis was conducted using the Fisher's exact test, and other categorical data were using the chi-square test. [c]

statistical analysis was conducted using the Mann-Whitney test. HAS, hepatoid adenocarcinoma of the stomach; AJCC, American Joint Committee on Cancer; AFP, alpha-fetoprotein; GEJ, gastroesophageal junction.

Table S17. Comparison of clinicopathological characteristics of HAS patients between ZJU-overall cohort and Liu et al. cohort

Characteristic	HAS		*P*
	ZJU-overall cohort (N = 90)	Liu et al. cohort (N = 55)	
**Age -years**			0.035
≤ 60	33(36.7)	30(54.5)	
> 60	57(63.3)	25(45.5)	
**Gender**			> 0.999
Female	24(26.7)	14(25.5)	
Male	66(73.3)	41(74.5)	
**T stage**			0.403
T1	7(10.0)	7(12.7)	
T2	15(21.4)	10(18.2)	
T3	26(37.1)	27(49.1)	
T4	22(31.4)	11(20.0)	
Unknown [a]	20	0	
**N stage**			0.371
N0	19(21.1)	10(18.2)	
N1	12(13.3)	16(29.1)	
N2	19(21.1)	15(27.3)	
N3	20(22.2)	14(25.5)	
Unknown [a]	20	0	
**M stage**			< 0.001[b]
M0	59(65.6)	54(98.2)	
M1	31(34.4)	1(1.8)	
**AJCC stage**			< 0.001
I	9(10.0)	8(14.5)	
II	16(17.8)	18(32.7)	
III	34(37.8)	28(50.9)	
IV	31(34.4)	1(1.8)	
**Location**			0.141
Antrum	43(47.8)	26(47.3)	
Body	22(24.4)	7(12.7)	
GEJ/Cardia	25(27.8)	22(40.0)	
**Serum AFP level -ng/mL**			0.005[c]
Median	201.5	20.9	
Range	0.9 – 41253.3	1.4 – 7335.0	
**Group by AFP -ng/mL**			0.002
≤ 20.0	21(23.3)	21(50.0)	
> 20.0	69(76.7)	21(50.0)	
Unknown [a]	0	13	

[a] The cases with unknown data were not included in the statistical analysis. [b] Statistical analysis was conducted using the Fisher's exact test, and other categorical data were using the chi-square test. [c]

statistical analysis was conducted using the Mann-Whitney test. HAS, hepatoid adenocarcinoma of the stomach; AJCC, American Joint Committee on Cancer; AFP, alpha-fetoprotein; GEJ, gastroesophageal junction.
